# Flotillin-2 dampens T cell antigen sensitivity and functionality

**DOI:** 10.1172/jci.insight.182328

**Published:** 2024-12-20

**Authors:** Sookjin Moon, Fei Zhao, Mohammad N. Uddin, Charles J. Tucker, Peer W.F. Karmaus, Michael B. Fessler

**Affiliations:** 1 Immunity, Inflammation and Disease Laboratory and; 2 Fluorescence Microscopy and Imaging Center, National Institute of Environmental Health Sciences (NIEHS), NIH, Research Triangle Park, North Carolina, USA.

**Keywords:** Immunology, Lipid rafts, T cell receptor, T cells

## Abstract

T cell receptor (TCR) engagement triggers T cell responses, yet how TCR-mediated activation is regulated at the plasma membrane remains unclear. Here, we report that deleting the membrane scaffolding protein Flotillin-2 (Flot2) increases T cell antigen sensitivity, resulting in enhanced TCR signaling and effector function in response to weak TCR stimulation. T cell–specific Flot2-deficient mice exhibited reduced tumor growth and enhanced immunity to infection. Flot2-null CD4^+^ T cells exhibited increased Th1 polarization, proliferation, Nur77 induction, and phosphorylation of ZAP70 and ERK1/2 upon weak TCR stimulation, indicating a sensitized TCR-triggering threshold. Single-cell RNA-Seq suggested that Flot2-null CD4^+^ T cells follow a similar route of activation as WT CD4^+^ T cells but exhibit higher occupancy of a discrete activation state under weak TCR stimulation. Given prior reports that TCR clustering influences sensitivity of T cells to stimuli, we evaluated TCR distribution with super-resolution microscopy. Flot2 ablation increased the number of surface TCR nanoclusters on naive CD4^+^ T cells. Collectively, we posit that Flot2 modulates T cell functionality to weak TCR stimulation, at least in part, by regulating surface TCR clustering. Our findings have implications for improving T cell reactivity in diseases with poor antigenicity, such as cancer and chronic infections.

## Abstract 2

Deletion of the membrane protein Flotillin-2 enhances T Cell Receptor signaling strength and T cell function.

## Graphical abstract

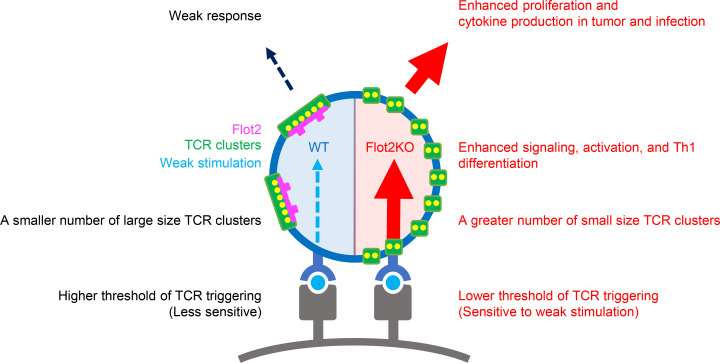

## Introduction

T cells encounter a vast array of antigens, but few cognate antigens induce T cell receptor (TCR) triggering and T cell activation. Proper regulation of TCR triggering plays a critical role in protective T cell immunity, but how T cells regulate TCR triggering remains unclear. The kinetic proofreading model, introduced in 1995, proposed that the duration of TCR interaction with the peptide-major histocompatibility complex (pMHC) is a critical determinant of TCR discrimination (1–6). By contrast, several studies have more recently suggested spatial organization of receptors on the membrane as a key determinant of TCR activation. Thus, in the engineered TCR signaling system, prolonged interaction alone is not sufficient to initiate TCR signaling; instead, receptor clustering on the membrane is necessary (7). Moreover, signal strength reportedly amplifies as the interligand spacing decreases (8), and the density of TCR-CD3 complexes within nanoclusters determines TCR triggering efficiency (9), together highlighting the importance of spatial thresholds in TCR triggering. Despite the accumulating evidence highlighting the role of surface TCR clustering in the initiation of TCR signaling, the regulation of TCR spatial organization on the membrane and its effect on fine-tuning of the TCR triggering threshold has remained elusive.

Flotillin-1 (Flot1) and Flot2 are evolutionarily conserved, ubiquitously expressed scaffolding proteins (10–15) that are thought to localize to lipid rafts, membrane microdomains that support receptor-mediated signaling (16–24). Rafts have been suggested to promote TCR signaling by supporting assembly of the immunological synapse and/or assisting signaling by costimulatory molecules (25, 26). Indeed, Flot2 has recently been implicated in TCRζ trafficking, but divergent findings have been reported on its role in TCR signaling, perhaps in part because of reliance on Jurkat T cell lines (27, 28). Overall, these conflicting observations underscore the need for further elucidation of the role of flotillins in T cell responses and their potential for therapeutic intervention.

Here, we investigated the role of Flot2 in TCR triggering and T cell responses using both in vivo and in vitro models. We found that Flot2-deficient mice exhibited delayed tumor growth and heightened resistance to *Listeria* infection, associated with augmented effector T cell proliferation and cytokine production. In vitro models revealed that Flot2-deficient CD4^+^ T cells exhibited enhanced TCR activation, even under weak TCR stimulation. Furthermore, Flot2-deficient CD4^+^ T cells showed heightened differentiation toward a Th1 subset upon exposure to various stimulus concentrations, including weak TCR stimulation. This enhanced differentiation is likely due to a sensitized TCR triggering threshold, as suggested by elevated phosphorylation of signaling proteins in the proximal TCR signaling pathway following weak stimulation. Single-cell RNA-Seq (scRNA-Seq) analysis suggested that *Flot2^CD4^* CD4^+^ T cells follow a similar route of activation as *Flot2^WT^* CD4^+^ T cells but exhibit higher occupancy in a discrete activation state under weak TCR stimulation. Finally, super-resolution imaging revealed an increased number of surface TCR nanoclusters on Flot2-deficient naive CD4^+^ T cells, suggesting that Flot2 controls spatial organization of TCR molecules in the steady-state and, thereby, sensitivity of naive T cells to the environment.

## Results

### Flot2 deletion enhances antitumor activity of T cells in murine tumor models.

**Figure 1:**
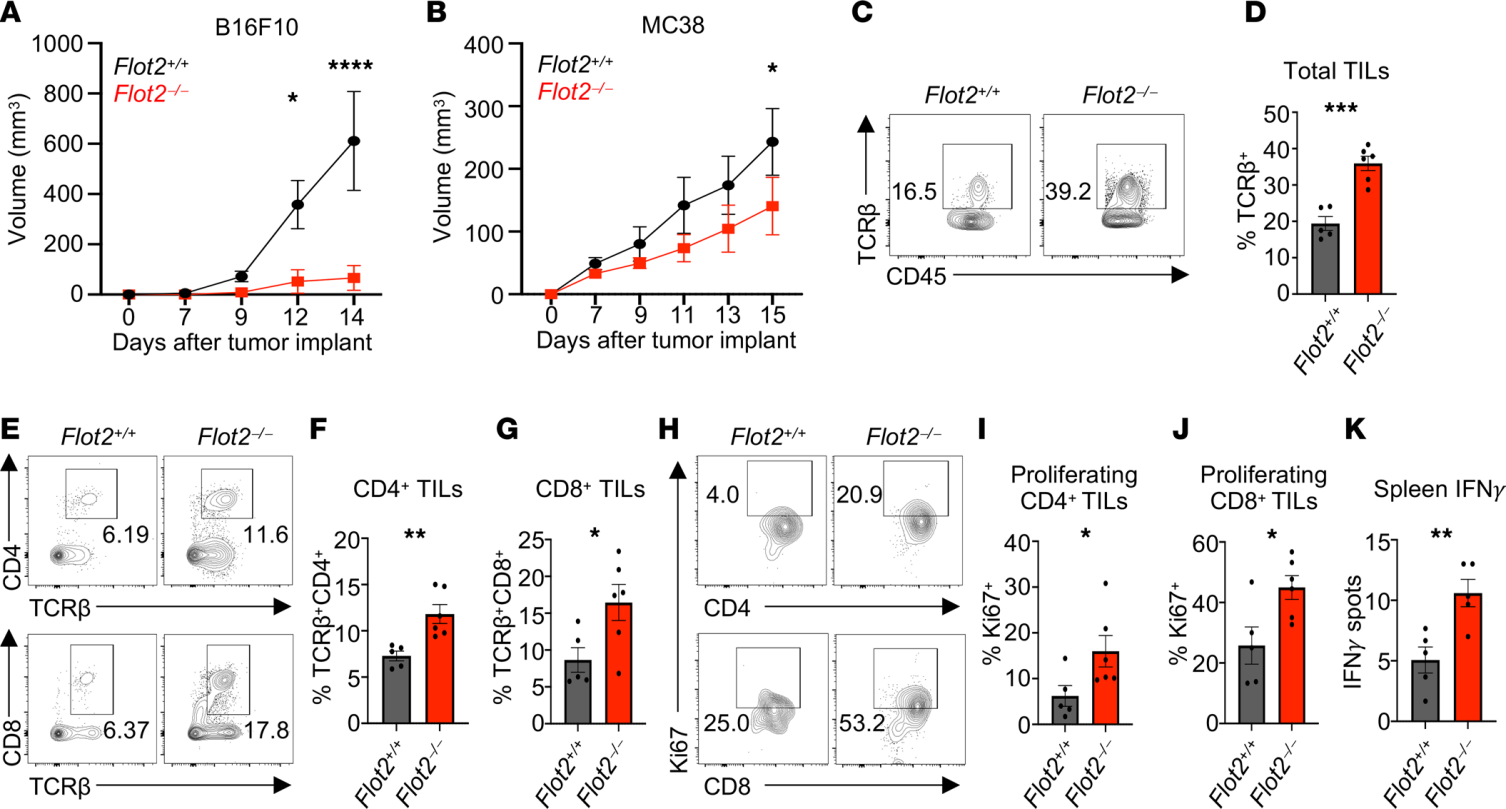
Flot2 deficiency potentiates the antitumor activity of both CD4^+^ and CD8^+^ T cells in vivo. (**A** and **B**) Tumor volume in *Flot2^+/+^* or *Flot2^–/–^* mice injected with B16F10 (**A**) or MC38 (**B**) (*n* = 6–7 per group). (**C**–**J**) Flow cytometric analysis of tumor-infiltrating lymphocytes (TILs) in B16F10 tumor–bearing *Flot2^+/+^* or *Flot2^–/–^* mice. Representative plots (**C**, **E**, and **H**) are shown. TCRβ^+^ (**D**), TCRβ^+^CD4^+^ (**F**), and TCRβ^+^CD8^+^ (**G**) populations within 7AAD^–^CD45^+^ population, and Ki67^+^ populations among 7AAD^–^CD45^+^TCRβ^+^CD4^+^CD44^+^CD62L^–^ population (**I**) or 7AAD^–^CD45^+^TCRβ^+^CD8^+^CD44^+^CD62L^–^ population (**J**), are depicted. (**K**) Splenocytes from B16F10 tumor–bearing *Flot2^+/+^* or *Flot2^–/–^* mice were stimulated with 1 μg/mL of TRP-2 melanoma peptide for 24 hours and assayed for antigen-specific reactivity using an IFN-γ ELISPOT assay. Data are representative of 2 independent experiments. Data were analyzed by unpaired *t* test (**D**, **F**, **G**, and **I**–**K**) or 2-way ANOVA followed with Šidák’s multiple-comparison tests (**A** and **B**). Data are shown as mean ± SEM; *P < 0.05; **P < 0.01; ***P < 0.001; ****P < 0.0001.

To investigate the role of Flot2 in T cell responses, we generated *Flot2* global KO mice (i.e., *Flot2^–/–^* mice) by flanking the *Flot2* coiled-coil domain (14) with loxP sites and then crossing these *Flot2^fl/fl^* mice with CMV-Cre mice (29) (Supplemental Figure 1; supplemental material available online with this article; https://doi.org/10.1172/jci.insight.182328DS1). B16F10 melanoma and MC38 colon adenocarcinoma, well-established in vivo models of T cell antitumor immunity (30–33), were first tested. In both the B16F10 and MC38 models, *Flot2^–/–^* mice exhibited delayed/reduced tumor growth compared with *Flot2*^+/+^ counterparts (Figure 1, A and B). Similar results were noted in mice of both sexes. Furthermore, *Flot2^–/–^* mice exhibited an increased frequency of CD4^+^ and CD8^+^ tumor-infiltrating lymphocytes (TILs) in the B16F10 melanoma model, specifically including Ki67^+^ proliferating effector CD4^+^ and CD8^+^ cells (Figure 1, C–J). The expression of TOX, a marker of functional exhaustion, was decreased in CD8^+^ TILs of *Flot2^–/–^* mice with MC38 tumors, whereas TIM-3 expression was similar to that in WT controls (Supplemental Figure 2, A–F). Total splenic IFN-γ production to melanoma TRP-2 peptide stimulation was also elevated in B16F10 tumor–bearing *Flot2^–/–^* mice compared with their *Flot2^+/+^* counterparts, indicating an enhanced response to tumor antigen (Figure 1K). Taken together, these results indicate an augmented antitumor immune response in Flot2-deficient mice.

Next, we sought to examine whether Flot2 deficiency specifically within the T cell compartment is sufficient to confer enhanced antitumor immunity. To explore this, we generated mixed bone marrow chimeras by reconstituting lethally irradiated TCRα-deficient (*TCR*α*^–/–^*) recipient mice with a 1:5 ratio mixture of bone marrow cells from either *Flot2^+/+^* or *Flot2^–/–^* donor mice and *TCR*α*^–/–^* mice, thereby generating mice with predominantly WT hematopoietic cells, except for a Flot2-deficient T cell compartment (34). Subsequently, these chimeras were inoculated with B16F10 tumors. Notably, *TCR*α*^–/–^* recipients reconstituted with *Flot2^–/–^* bone marrow exhibited a significant reduction in tumor volumes compared with those reconstituted with WT bone marrow (Supplemental Figure 2G). Furthermore, we observed increased proliferation marker Ki67 expression in both CD4^+^ and CD8^+^ TILs in the chimeras transferred with *Flot2^–/–^* bone marrow, along with an expansion of the CD44^+^IFN-γ^+^ population within CD8^+^ TILs (Supplemental Figure 2, H–J). These findings suggest that Flot2 deficiency specifically within T cells augments anticancer immune responses.

This finding prompted us to generate T cell–specific Flot2-deficient mice through crossbreeding of *Flot2^fl/fl^* mice with *CD4^Cre^* mice (i.e., *Flot2^CD4^* mice) in order to further explore the T cell–intrinsic role of Flot2 in anticancer immunity. Selective Flot2 deletion in T cells (Supplemental Figure 3, A–C) did not cause overt abnormalities in thymocyte development (Supplemental Figure 3, D–H). In the steady state, peripheral T cells in the lymph nodes of *Flot2^CD4^* mice exhibited similar characteristics to those in *Flot2^WT^* mice. There was, however, a marginal decrease in total CD4^+^ T cell numbers, accompanied by an increase in the percentage of the CD44^+^CD62L^–^ population, as well as enhanced expression of Nur77, T-bet, and LFA-1α within CD4^+^ T cells, suggesting a shift from naive to activated status (Supplemental Figure 3, I–N).

**Figure 2:**
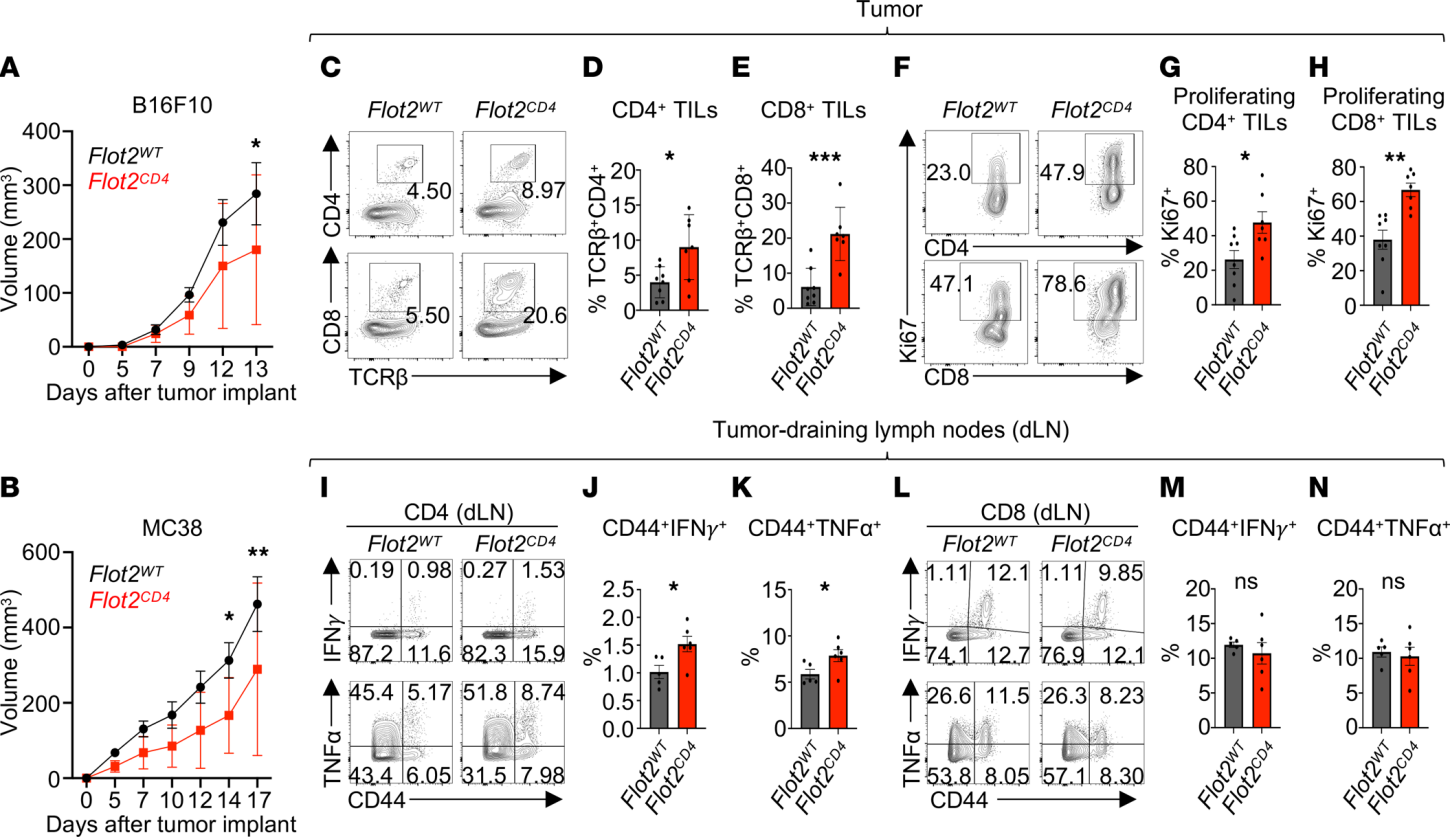
T cell–specific Flot2 deficiency potentiates the antitumor activity of both CD4^+^ and CD8^+^ T cells in vivo. (**A** and **B**) Tumor volume in *Flot2^WT^* or *Flot2^CD4^* mice injected with B16F10 (**A**; *n* = 10 per group) or MC38 (**B**, *n* = 9 for *Flot2^WT^* and *n* = 14 for *Flot2^CD4^*). (**C**–**H**) Flow cytometric analysis of TILs. Representative plots (**C** and **F**) are shown. TCRβ^+^CD4^+^ (**D**) and TCRβ^+^CD8^+^ (**E**) populations among 7AAD^–^CD45.2^+^ population and Ki67^+^ populations among 7AAD^–^CD45.2^+^TCRβ^+^CD4^+^ population (**G**) or 7AAD^–^CD45.2^+^TCRβ^+^CD8^+^ population (**H**) in B16F10-bearing *Flot2^WT^* or *Flot2^CD4^* mice are presented. (**I**–**N**) Flow cytometric analysis of tumor-draining lymph nodes (dLNs). Representative plots (**I** and **L**) are displayed. CD44^+^IFN-γ^+^ (**J**) and CD44^+^TNF-α^+^ (**K**) populations among 7AAD^–^CD45.2^+^TCRβ^+^CD4^+^ population and CD44^+^IFN-γ^+^ (**M**) and CD44^+^TNF-α^+^ (**N**) among 7AAD^–^CD45.2^+^TCRβ^+^CD8^+^ population in B16F10-bearing *Flot2^WT^* or *Flot2^CD4^* mice are indicated. Data are representative of 2 independent experiments (**A**–**N**). Data were analyzed by unpaired *t* test (**D**, **E**, **G**, **H**, **J**, **K**, **M**, and **N**) or 2-way ANOVA followed with Šidák’s multiple-comparison tests (**A** and **B**). Data are shown as mean ± SEM; *P < 0.05; **P < 0.01; ***P < 0.001.

As above for *Flot2^–/–^* mice, *Flot2^CD4^* mice were evaluated in the B16F10 melanoma and MC38 colon adenocarcinoma models. Consistent with the phenotypes of *Flot2^–/–^* mice, *Flot2^CD4^* mice showed reduced growth of both B16F10 and MC38 tumors compared with *Flot2^WT^* controls (Figure 2, A and B). *Flot2^CD4^* mice also showed elevated populations of CD4^+^ and CD8^+^ TILs, as well as increased Ki67^+^ proliferating effector CD4^+^ and CD8^+^ cells in the B16F10 melanoma model (Figure 2, C–H). Moreover, we observed heightened expression of IFN-γ and TNF-α effector cytokines in CD4^+^ T cells within the tumor-draining lymph nodes (dLN) of *Flot2^CD4^* mice (Figure 2, I–K). However, no discernible difference was observed in CD8^+^ T cells within the dLN (Figure 2, L–N). Overall, these data demonstrate that specific Flot2 deficiency in T cells boosts antitumor responses of both CD4^+^ and CD8^+^ T cells in murine tumor models.

### Flot2 deficiency boosts the antibacterial responses of CD4^+^ and CD8^+^ T cells in vivo.

**Figure 3:**
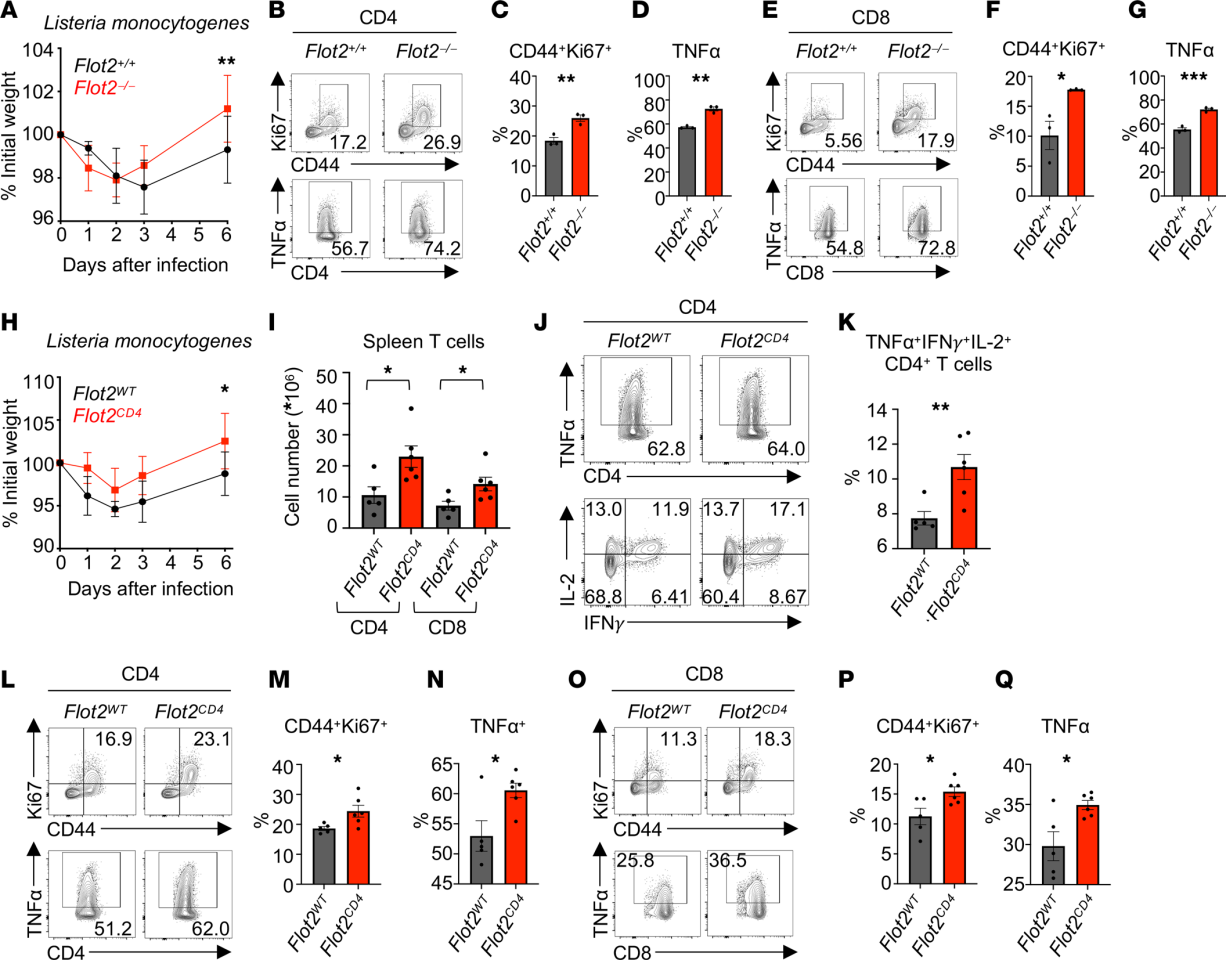
Flot2 deficiency promotes CD4^+^ and CD8^+^ T cell responses against *Listeria monocytogenes* infection. (**A**) Weight loss in *Flot2^+/+^* or *Flot2^–/–^* mice following *Listeria monocytogenes* infection (5,000 CFU per mouse) is presented as the mean percentage of initial weight (*n* = 4–5 per group). (**B**–**G**) Flow cytometric analysis of splenic T cells from *Listeria*-infected *Flot2^+/+^* or *Flot2^–/–^* mice. Representative plots (**B** and **E**) are provided. CD44^+^Ki67^+^ (**C**) and TNF-α^+^ (**D**) populations within viable CD45^+^TCRβ^+^CD4^+^ population and CD44^+^Ki67^+^ (**F**) and TNF-α^+^ (**G**) populations within viable CD45^+^TCRβ^+^CD8^+^ population are shown. (**H**) Weight loss in *Flot2^WT^* or *Flot2^CD4^* mice following *L. monocytogenes* infection (5,000 CFU per mouse) is presented as the mean percentage of initial weight (*n* = 5–6 per group). (**I**) Splenic CD4^+^ and CD8^+^ T cells numbers in infected *Flot2^WT^* or *Flot2^CD4^* mice are depicted. (**J**–**Q**) Flow cytometric analysis of splenic T cells from *L. monocytogenes*–infected *Flot2^WT^* or *Flot2^CD4^* mice. Representative plots (**J**, **L,** and **O**) are shown. TNF-α^+^IFN-γ^+^IL-2^+^ (**K**), CD44^+^Ki67^+^ (**M**), and TNF-α^+^ (**N**) populations within viable CD45^+^TCRβ^+^CD4^+^ population and CD44^+^Ki67^+^ (**P**) and TNF-α^+^ (**Q**) populations within viable CD45^+^TCRβ^+^CD8^+^ population are shown. Data are representative of 2 independent experiments (**A**–**Q**). Data were analyzed by unpaired *t* test (**C**, **D**, **F, G, I**, **K**, **M**, **N**, **P**, and **Q**) or 2-way ANOVA followed with Šidák’s multiple-comparison tests (**A** and **H**). Data are shown as mean ± SEM; *P < 0.05; **P < 0.01; ***P < 0.001.

To evaluate the role of Flot2 in regulating antibacterial immune responses in vivo, we next challenged mice with *Listeria monocytogenes*. Following infection, *Flot2^–/–^* mice showed elevated resistance to weight loss compared with *Flot2^+/+^* mice (Figure 3A). Furthermore, both CD4^+^ and CD8^+^ T cells in *Flot2^–/–^* mice exhibited increased expression of Ki67 and TNF-α compared with *Flot2^+/+^* mice (Figure 3, B–G), suggesting increased proliferation and effector function. Consistent with this, CD44^+^T-bet^+^, CD44^+^IFN-γ^+^, CD44^–^TNF-α^+^, and CD44^+^IL-2^+^ populations were all augmented in both CD4^+^ and CD8^+^ splenic T cells (Supplemental Figure 4). Given these findings, we next used *Flot2^CD4^* mice to investigate whether T cell–specific Flot2 deficiency also improves the antibacterial T cell response. Notably, *Flot2^CD4^* mice also showed less weight loss compared with *Flot2^WT^* after *L*. *monocytogenes* infection (Figure 3H). Quantification of the absolute number of T cells in the spleen of infected *Flot2^WT^* and *Flot2^CD4^* mice revealed an increase in both CD4^+^ and CD8^+^ T cell numbers in *Flot2^CD4^* mice (Figure 3I). Moreover, *Flot2^CD4^* mice had increased TNF-α^+^IFN-γ^+^IL-2^+^ multifunctional CD4^+^ T cells, which play a crucial role in infection control (35, 36) (Figure 3, J and K). Consistent with findings in *Flot2^–/–^* mice, *Flot2^CD4^* mice also exhibited heightened expression of Ki67 and TNF-α in both CD4^+^ and CD8^+^ splenic T cells (Figure 3, L–Q). These data collectively suggest that Flot2 deficiency augments the responses of both CD4^+^ and CD8^+^ T cells in the context of in vivo infection with *L*. *monocytogenes*.

### TCR activation induces enhanced response in Flot2-deficient CD4^+^ but not CD8^+^ T cells.

**Figure 4:**
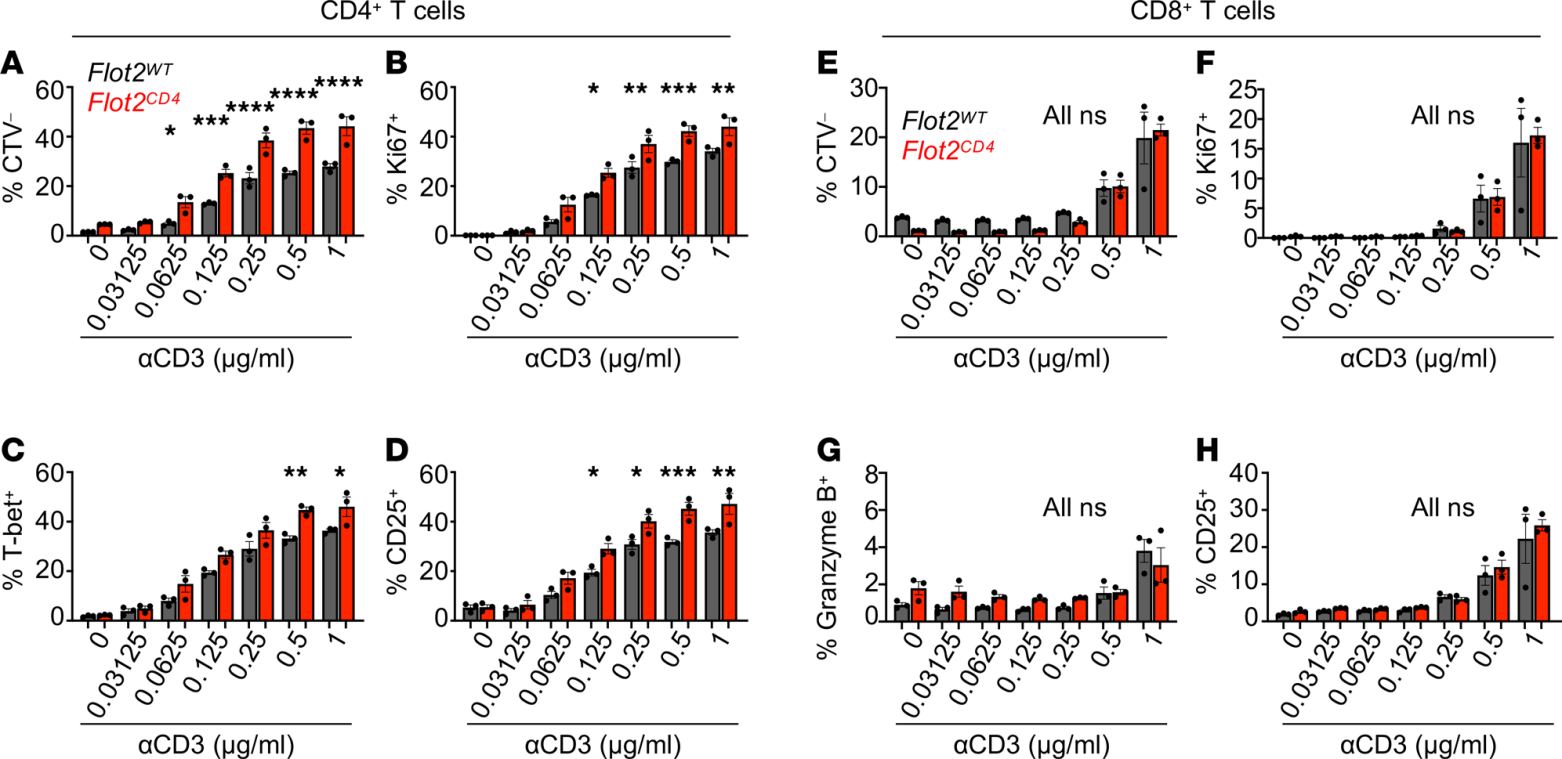
*Flot2^CD4^* CD4^+^ T cells, but not *Flot2^CD4^* CD8^+^ T cells, showed enhanced T cell responses during in vitro T cell stimulation. (**A**–**D**) Naive CD4^+^ T cells were purified and stimulated in vitro for 72 hours with varying doses of plate-bound αCD3, alongside a fixed dose of soluble αCD28 (1 μg/mL), followed by flow cytometric analysis to assess cell proliferation and activation. CTV^–^ (**A**), Ki67^+^ (**B**), T-bet^+^ (**C**), and CD25^+^ (**D**) populations within viable TCRβ^+^CD4^+^ population are shown. (**E**–**H**) Naive CD8^+^ T cells were purified and stimulated in vitro for 72 hours with varying doses of plate-bound αCD3, alongside a fixed dose of soluble αCD28 (1 μg/mL), followed by flow cytometric analysis to assess cell proliferation and activation. CTV^–^ (**E**), Ki67^+^ (**F**), Granzyme B^+^ (**G**), and CD25^+^ (**H**) populations within viable TCRβ^+^CD8^+^ population are shown. Data are representative of 2 independent experiments (**A**–**H**). Data were analyzed by 1-way ANOVA followed with Šidák’s multiple-comparison tests (**A**–**H**). Data are shown as mean ± SEM; *P < 0.05; **P < 0.01; ***P < 0.001; ****P < 0.0001.

After confirming that Flot2 deficiency enhances effector T cell responses in vivo in both tumor and infection models, we investigated this phenomenon mechanistically, using reductionist in vitro approaches. Purified naive CD4^+^ and CD8^+^ T cells from *Flot2^WT^* or *Flot2^CD4^* mice were stimulated with increasing concentrations of plate-bound αCD3, along with a fixed concentration of soluble αCD28 (1 μg/mL), modeling TCR stimulation and costimulation exclusively. Following stimulation, both *Flot2^WT^* and *Flot2^CD4^* CD4^+^ T cells showed a concentration-dependent increase in CellTrace Violet^–^ (CTV^–^; i.e., proliferated), Ki67^+^, T-bet^+^, and CD25^+^ populations, confirming T cell activation in line with the strength of TCR stimulation (Figure 4, A–D, and Supplemental Figure 5A). *Flot2^CD4^* CD4^+^ T cells exhibited heightened proliferation (CTV^–^ and Ki67^+^) and T-bet and CD25 expression compared with *Flot2^WT^* across various concentrations of αCD3 (Figure 4, A–D, and Supplemental Figure 5A). Remarkably, Flot2 deficiency augmented CD4^+^ T cell proliferation even at very low concentrations of αCD3 (0.0625 μg/mL) (Figure 4A) and increased expression of the early T cell activation marker (CD25) at low concentrations of αCD3 (0.125 μg/mL) (Figure 4D), highlighting enhanced T cell responsiveness to weak TCR stimulation. By contrast, Flot2-deficient CD8^+^ T cells did not experience increased cell proliferation or early activation compared with WT controls (Figure 4, E–H, and Supplemental Figure 5B). Collectively, these findings indicate that Flot2 deficiency renders CD4^+^ T cells more responsive to weak TCR stimulation in the presence of both TCR stimulation and costimulation, whereas Flot2-deficient CD8^+^ T cells may rely on supplemental factors, potentially available in vivo, for their boosted activation.

### Deletion of Flot2 in CD4^+^ T cells promotes Th1 cell differentiation.

**Figure 5:**
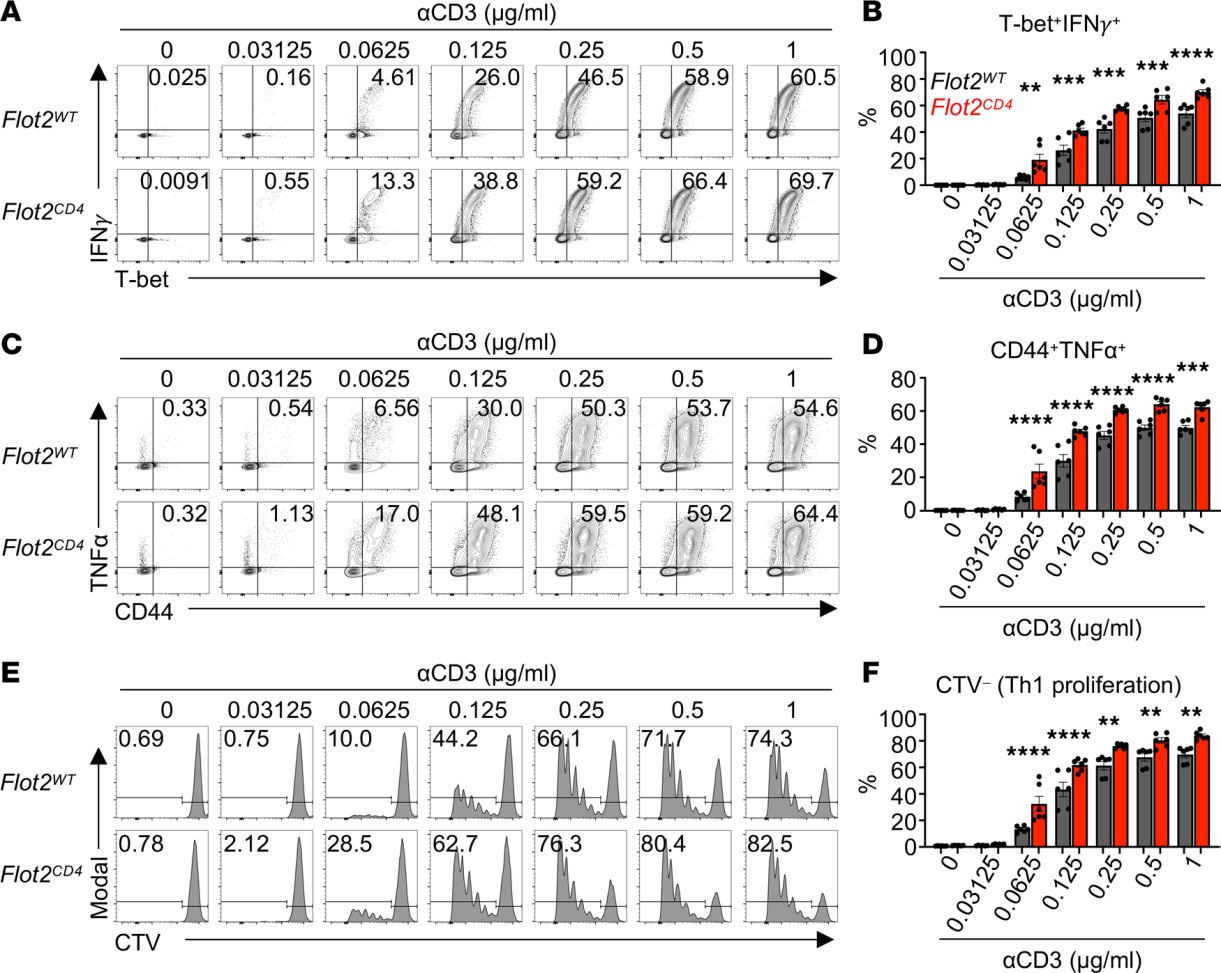
Flot2 ablation promotes CD4^+^ T cell differentiation into Th1 upon weak TCR stimulation. (**A**–**F**) Naive CD4^+^ T cells were purified and differentiated toward the Th1 subtype in vitro using Th1 polarizing conditions, followed by flow cytometric analysis to assess Th1 polarization, cytokine production, and cell proliferation. Representative plots (**A**, **C**, and **E**) are shown. T-bet^+^IFN-γ^+^ (**B**), CD44^+^TNF-α^+^ (**D**), and CTV^–^ (**F**) populations within viable TCRβ^+^CD4^+^ population are shown. Data are representative of 3 independent experiments (**A**–**F**). Data were analyzed by 1-way ANOVA followed with Šidák’s multiple-comparison tests (**B**, **D**, and **F**). Data are shown as mean ± SEM; **P < 0.01; ***P < 0.001; ****P < 0.0001.

During T cell activation, naive CD4^+^ T cells have the potential to differentiate into various Th cell subsets, characterized by distinct transcription factors, cytokines, and functions (37–39). The determination of cell fate during differentiation is influenced by both TCR signal strength and the cytokine milieu, with recent findings suggesting an association between strong TCR signals and Th1 differentiation (40–42). Since in vitro stimulated Flot2-deficient CD4^+^ T cells express higher levels of CD25 and T-bet (Figure 4, C and D), indicative of strong TCR signal strength, we investigated the effect of Flot2 deficiency on Th differentiation. Initially, naive CD4^+^ T cells were differentiated in vitro using Th1 polarizing medium and varying concentration of plate-bound αCD3. Both *Flot2^WT^* and *Flot2^CD4^* CD4^+^ T cells exhibited concentration-dependent induction of Th1 polarization and proliferation (Figure 5). Notably, *Flot2^CD4^* CD4^+^ T cells displayed a significant increase in T-bet^+^IFN-γ^+^ and CD44^+^TNF-α^+^ populations compared with *Flot2^WT^* CD4^+^ T cells across various concentrations of αCD3, even at very low concentrations (Figure 5, A–D). Furthermore, Th1 cell proliferation of *Flot2^CD4^* CD4^+^ T cells was also robustly induced at low concentrations of αCD3, likely due to their increased sensitivity to TCR stimulation (Figure 5, E and F).

Next, we evaluated differentiation into other Th subsets using Th2, Th17, or Treg polarizing conditions. In contrast to Th1 differentiation, *Flot2^CD4^* CD4^+^ T cells showed no difference in Th2 proliferation, evidenced by the CTV^–^ population, and a decrease in IL-4^+^GATA3^+^ populations at sufficient TCR stimulation (Supplemental Figure 6, A–D). Similarly, no difference was observed in Th17 differentiation (Supplemental Figure 6, E and F). However, *Flot2^CD4^* CD4^+^ T cells showed augmented differentiation into Foxp3^+^ Treg populations selectively upon weak TCR stimulation (Supplemental Figure 6, G and H). Given that Treg differentiation tends to favor low-abundance, high-affinity antigens (43), this finding may reflect the increased reactivity of *Flot2^CD4^* CD4^+^ T cells to the low abundance of αCD3 compared with *Flot2^WT^* CD4^+^ T cells. On the basis of these findings, we conclude that Flot2-deficient CD4^+^ T cells are inclined toward Th1 and Treg differentiation even at very low concentrations of αCD3, likely due to their hypersensitivity to weak TCR stimulation.

### Flot2 ablation decreases TCR triggering threshold in CD4^+^ T cells.

**Figure 6:**
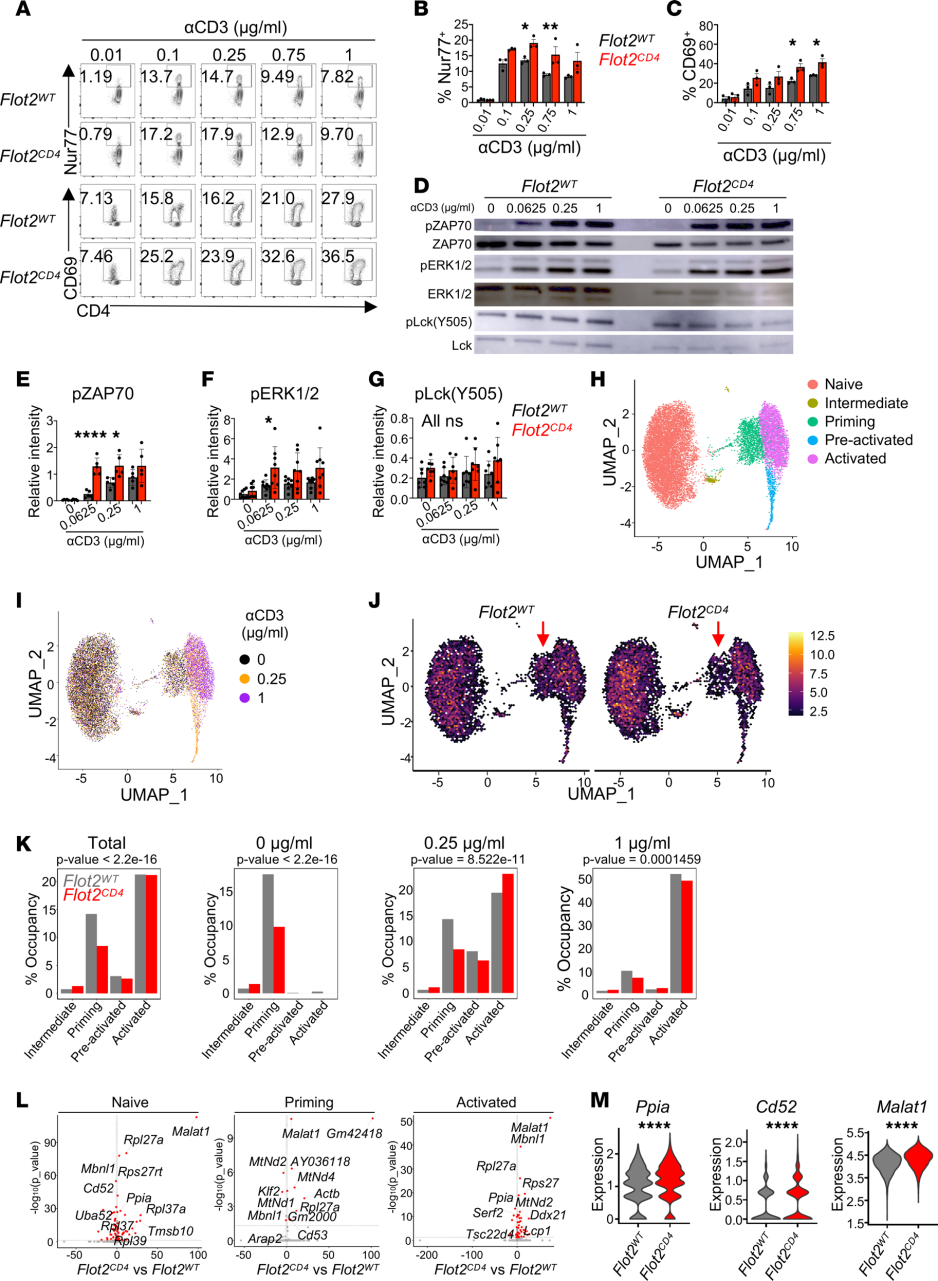
Flot2 ablation sensitizes TCR triggering threshold in CD4^+^ T cells. (**A**–**C**) Naive CD4^+^ T cells were stimulated in vitro for 3 hours (**B**) or 24 hours (**C**) with varying doses of plate-bound αCD3, alongside a fixed dose of soluble αCD28 (1 μg/mL), followed by FACS. Representative plots (**A**) show Nur77^+^ (**B**) and CD69^+^ (**C**) populations within viable TCRβ^+^CD4^+^ population. (**D**–**G**) Western blot of the phosphorylation of TCR signaling molecules in naive CD4^+^ T cells stimulated with varying doses of plate-bound αCD3 for 3 minutes. Representative blots (**D**) and quantifications of pZAP70 (**E**), pERK1/2 (**F**), and pLck (Y505) (**G**), normalized to their respective total protein levels (ZAP70, ERK1/2, and Lck), are shown. (**H** and **I**) scRNA-Seq was performed on naive CD4^+^ T cells following a 3-hour stimulation with varying concentrations of plate-bound αCD3. A fixed dose of soluble αCD28 (1 μg/mL) was provided under the conditions of 0.25 or 1 μg/mL of plate-bound αCD3. Unsupervised T cell clusters were annotated as 5 distinguishable functional states on a UMAP plot (**H**), and the effect of stimulation dose was projected onto the UMAP (**I**). (**J**) Distribution of *Flot2^WT^* or *Flot2^CD4^* CD4^+^ T cells in each cluster. Red arrows indicate the priming clusters for each genotype. (**K**) Occupancy of *Flot2^WT^* or *Flot2^CD4^* genotype in each T cell cluster, analyzed by total or each stimulatory condition. (**L**) Volcano plots of spliced RNA in naive, priming, and activated clusters. (**M**) Expression level of spliced RNA related to cellular proliferation in the naive cluster. Data are pooled from 3 (**A**–**C**), 5 (**E**), 8 (**F**), or 7 (**G**) independent experiments. Data were analyzed by 1-way ANOVA followed with Šidák’s multiple-comparison tests (**B**, **C**, and **E**–**G**), χ^2^ analysis (**K**), or Wilcoxon ranked-sum test (**M**). Data are shown as mean ± SEM; *P < 0.05; **P < 0.01; ****P < 0.0001.

Given the heightened proliferation, activation, and Th1 differentiation observed in Flot2-deficient CD4^+^ T cells, we hypothesized an augmentation in TCR signaling in CD4^+^ T cells upon Flot2 ablation. To test this, we stimulated purified naive CD4^+^ T cells from *Flot2^WT^* or *Flot2^CD4^* mice with varying concentrations of plate-bound αCD3 in vitro and analyzed them after 3 hours or 24 hours to observe early-phase T cell activation. *Flot2^CD4^* CD4^+^ T cells exhibited elevated expression of Nur77, a marker of TCR signal strength, particularly at low concentrations of αCD3 (Figure 6, A and B). Additionally, the early T cell activation marker CD69 was increased in *Flot2^CD4^* CD4^+^ T cells compared with *Flot2^WT^* CD4^+^ T cells (Figure 6, A and C). Conversely, *Flot2^CD4^* CD8^+^ T cells did not exhibit enhanced expression of Nur77 and CD69, further emphasizing the necessity of additional factors for the Flot2-dependent activation phenotype in CD8^+^ T cells (Supplemental Figure 7, A–C). We next investigated earlier phases of TCR signaling by profiling phosphorylation of TCR signaling molecules after 3 minutes of in vitro stimulation. Notably, early phosphorylation of ZAP70 and ERK1/2 induced by TCR triggering was enhanced in *Flot2^CD4^* CD4^+^ T cells compared with *Flot2^WT^* CD4^+^ T cells under weak stimulation (Figure 6, D–F). Meanwhile, the phosphorylation of Lck at Y505, an inactivating phosphorylation, showed no difference between the 2 genotypes (Figure 6, D and G). Altogether, these data show enhanced signaling and early activation of *Flot2^CD4^* CD4^+^ T cells compared with *Flot2^WT^* CD4^+^ T cells upon suboptimal stimulation, indicating that Flot2 ablation lowers the TCR triggering threshold.

To gain a more comprehensive understanding of the role of Flot2 in CD4^+^ T cells during T cell activation, we next performed scRNA-Seq on naive *Flot2^WT^* or *Flot2^CD4^* CD4^+^ T cells after in vitro stimulation with varying concentrations of αCD3 antibody–mediated TCR stimulation: no (0 μg/mL), weak (0.25 μg/mL), or strong (1 μg/mL) stimulation for 3 hours. Clustering the results using the Leiden algorithm revealed 5 distinct functional states based on expression of marker genes — naive, intermediate, priming, preactivated, and activated — as visualized using Uniform Manifold Approximation and Projection (UMAP) (Figure 6H and Supplemental Figure 7D). These clusters aligned well with previously reported gene sets related to early T cell activation (44) and hallmark genes of T cell activation (*Nr4a1*, *Myc*, *Cd69*, *Il2ra*), and naive status (*Tcf7*, *Ccr7*, *Cd4*, *Sell*) (Supplemental Figure 7, E–G). The integration of stimulation concentrations into the UMAP plot revealed that cells from the 0 μg/mL group were mostly in the naive cluster and cells from the 1 μg/mL group were mostly in the activated cluster, validating our analysis (Figure 6I). Notably, naive CD4^+^ T cells exposed to weak stimulation (0.25 μg/mL) were distributed across diverse clusters spanning from naive to activated states, indicating that reducing the αCD3 concentration led to increased heterogeneity in the transcriptomic profile during T cell activation (Figure 6I). Interestingly, the distribution of the 2 genotypes significantly differed across activation clusters, with *Flot2^CD4^* CD4^+^ T cells showing a significantly lower frequency in the priming cluster (red arrow) compared with *Flot2^WT^* CD4^+^ T cells (Figure 6, J and K). By contrast, *Flot2^CD4^* CD4^+^ T cells demonstrated a higher occupancy in the activated cluster following weak stimulation (0.25 μg/mL) compared with *Flot2^WT^* CD4^+^ T cells, consistent with flow cytometric analysis following in vitro stimulation (Figure 6, B and K).

Next, using RNA velocity analysis, we examined if Flot2 deficiency affected T cell activation trajectories following in vitro stimulation. Our observations revealed nearly overlapping RNA velocity UMAP space between *Flot2^WT^* and *Flot2^CD4^* CD4^+^ T cells, indicating that both groups undergo similar major transcriptional changes during the early stages of activation, regardless of Flot2 expression (Supplemental Figure 7, H–J). Although *Flot2^WT^* and *Flot2^CD4^* CD4^+^ T cells displayed similar transcriptomic changes throughout activation, our analysis revealed increased spliced RNA expression of genes associated with cellular proliferation in *Flot2^CD4^* CD4^+^ T cells (Figure 6, L and M). Specifically, even in the naive state, *Flot2^CD4^* CD4^+^ T cells demonstrated higher expression levels of spliced *Ppia*, *Cd52*, and *Malat1* (Figure 6M). These genes are known to positively regulate T cell proliferation and activation as well as to enhance cytotoxic T cell differentiation and cytokine production (45–50). This suggests that while gene expression alterations remain consistent, differences in isoform usage may contribute to variations in T cell activation between *Flot2^WT^* and *Flot2^CD4^* CD4^+^ T cells.

Collectively, these findings suggest that Flot2 deficiency lowers the TCR triggering threshold in CD4^+^ T cells, resulting in enhanced TCR signaling and activation, particularly in response to weak TCR stimulation. Flot2 deficiency does not alter the intrinsic activation trajectory of CD4^+^ T cells but appears to alter the occupancy of a priming state, promoting the progression from the naive to the fully activated state upon weak stimulation.

### Flot2 controls TCR nanoclustering on the plasma membrane of naive CD4^+^ T cells.

**Figure 7:**
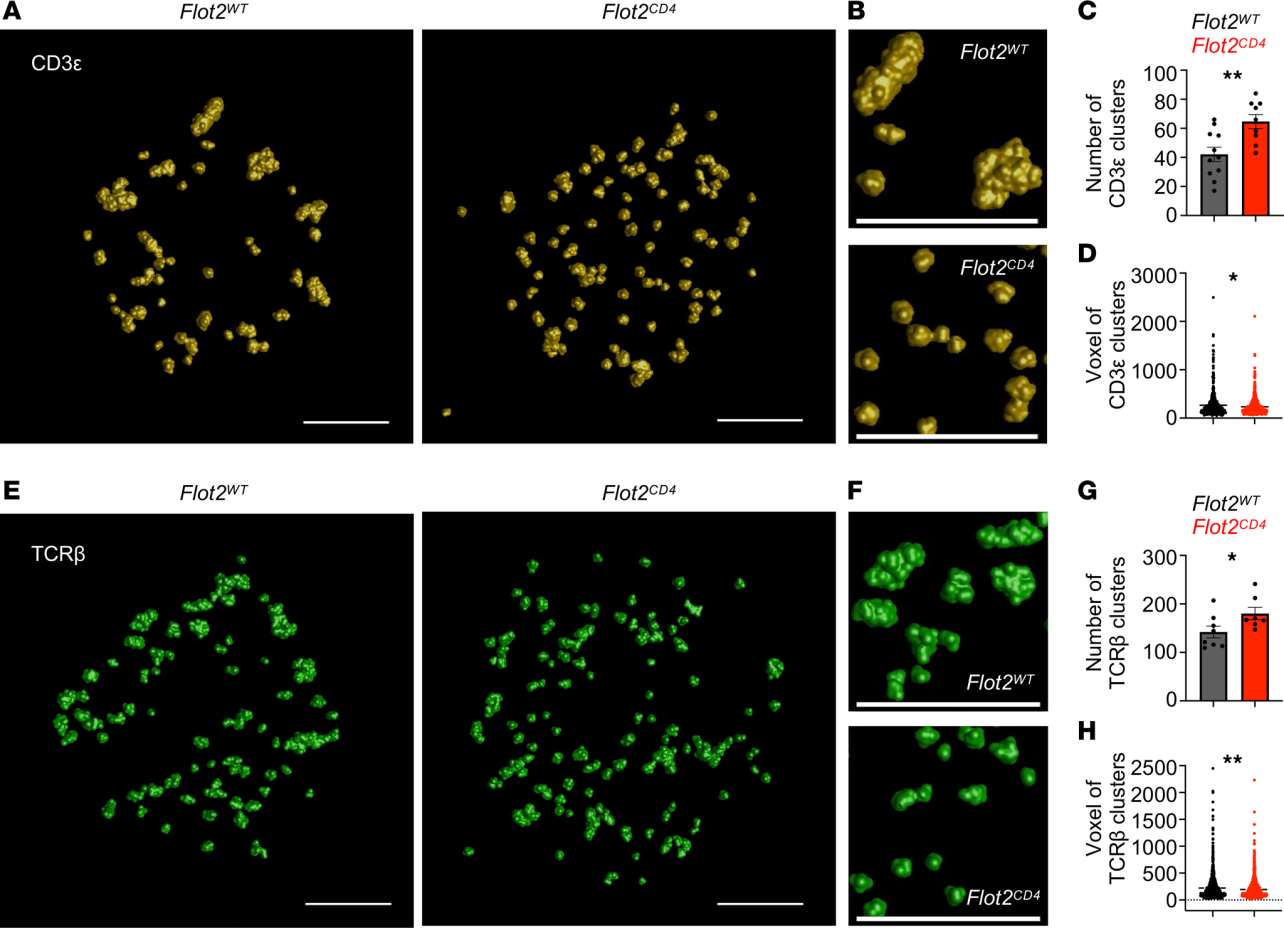
Flot2 ablation increases the number of surface TCR nanoclusters with a smaller size on naive CD4^+^ T cells. (**A**–**H**) dSTORM analysis of TCR nanoclustering in *Flot2^WT^* and *Flot2^CD4^* naive CD4^+^ T cells. Clustering images of CD3ε molecules (**A** and **B**) or TCRβ molecules (**E** and **F**) are shown. Number of clusters and voxel of CD3ε (**C** and **D**) or TCRβ (**G** and **H**) molecules after quantification using Huygens Cluster Analyzer are depicted. Scale bars: 1 μm. Data are representative of 4 (**A**–**D**) or 2 (**E**–**H**) independent experiments. Data were analyzed by unpaired *t* test (**C**, **D**, **G**, and **H**). Data are shown as mean ± SEM; *P < 0.05; **P < 0.01.

Receptor clustering is pivotal for setting thresholds in various signaling pathways (51, 52). While TCRs form nanoclusters and their clustering is crucial for TCR signaling regulation (7–9, 53–56), the mechanisms governing TCR nanoclustering remain unclear. Based on our findings demonstrating a role of Flot2 in regulating TCR signaling initiation, we hypothesized that Flot2 may also regulate TCR nanoclustering. Utilizing super-resolution imaging, we examined TCR nanoclusters in steady-state naive CD4^+^ T cells from *Flot2^WT^* or *Flot2^CD4^* mice, identifying them with CD3ε or TCRβ markers as previously described (9, 57). Notably, we found that naive *Flot2^CD4^* CD4^+^ T cells exhibited a higher number of CD3ε^+^ TCR nanoclusters in the steady state (Figure 7, A–C). Associated with this was a reduction in cluster size, as measured using volumetric space (voxel) analysis (Figure 7D). This increased number of small clusters resulted in a pattern of scattered TCR clusters on the membrane, giving the appearance of greater overall coverage (Figure 7A). Consistent with CD3ε^+^ nanocluster analysis, there was an increase in the number of TCRβ^+^ nanoclusters of smaller sizes (Figure 7, E–H). Convex hull geometry analysis further confirmed reduced volume, surface, the largest length, and the largest width of the convex hull of the cluster in *Flot2^CD4^* naive CD4^+^ T cells compared with *Flot2^WT^* counterparts (Supplemental Figure 8). In summary, these results suggest that Flot2 ablation regulates TCR nanoclustering by promoting the formation of an increased number of small clusters on the plasma membrane of naive CD4^+^ T cells.

## Discussion

T cell immunity relies on the proper initiation of TCR signaling, but how TCR triggering is regulated on the plasma membrane remains unclear. A few prior reports have implicated flotillins in TCR signaling, but conflicting findings have been noted, likely due to contextual factors such as the varying cell types (primary or cell line), stimulation methods, and experimental models that have been used (27, 28, 58). In the present study, we employed complementary in vivo and in vitro models of primary CD4^+^ and CD8^+^ T cells and included titrated TCR stimulation to comprehensively profile the role of Flot2 in regulating T cell effector functions, T cell differentiation, T cell activation, TCR triggering, and surface TCR distribution. Through this array of approaches, we have identified that Flot2 regulates the TCR triggering threshold and T cell functional responses in CD4^+^ T cells, potentially by orchestrating the spatial organization of surface TCR molecules (Supplemental Figure 9).

An intriguing observation comparing our in vivo and ex vivo model systems was that *Flot2^CD4^* CD4^+^ T cells displayed an augmented activation profile consistent with the in vivo setting, whereas *Flot2^CD4^* CD8^+^ T cells exhibited no noticeable alteration upon ex vivo stimulation. These findings imply that CD8^+^ T cells, unlike CD4^+^ T cells, may depend on additional in vivo signals to demonstrate a Flot2-dependent enhanced activation phenotype. The traditional concept of T cell activation encompasses the activation of both CD4^+^ and CD8^+^ T cells via TCR stimulation (signal 1) and costimulation (signal 2) (59, 60). It remains unclear to what extent distinct regulatory mechanisms govern TCR triggering in CD4^+^ versus CD8^+^ T cells, although a few prior reports have identified some cell type–specific differences, including in the effect of IL-2 on TCR signaling threshold and in the reliance of TCR signaling upon select adaptor proteins (61, 62). Given that Flot2 regulates ganglioside trafficking from the plasma membrane (63), and CD4^+^ and CD8^+^ T cells reportedly have different lipid raft ganglioside composition (64) as well as differential dependence on specific gangliosides for TCR activation (65), it is possible that Flot2 differentially affects signaling in the 2 T cell subsets via effects on membrane lipids. It is also possible that CD8^+^ T cells require supplemental Flot2-interacting cues, such as alternative costimulatory signals, integrin engagements, or cytokine effects, during their interactions with antigen-presenting cells. Exploring potential divergent regulatory mechanisms in the initial TCR triggering of CD4^+^ and CD8^+^ T cells presents an interesting avenue for future investigation. Such studies may have important translational value, given that CD4^+^ and CD8^+^ chimeric antigen receptor–expressing T cells used in cancer therapy have distinct functional responses to TCR stimulation (66).

A previous study demonstrated that the strength of stimulation does not inherently determine the transcriptional pathway of activated T cells; rather, it regulates the speed and synchronicity of cellular activation initiation (44). Considering this, the observation that *Flot2^CD4^* CD4^+^ T cells experienced stronger TCR signaling upon weak stimulation, while overall transcriptional pathways were comparable with WT, suggests that Flot2 deficiency might expedite the transition of cells from a naive state to full activation, rather than altering the T cell activation pathway. This interpretation is further supported by the reciprocal reduced and increased occupancy of *Flot2^CD4^* CD4^+^ T cells in the priming and activation clusters, respectively, upon weak stimulation. Additionally, the comparable transcriptional activation route suggests that Flot2-mediated regulation of TCR triggering might entail a distinct mechanism, potentially associated with protein spatial organization rather than gene expression regulation. This interpretation is supported by prior reports demonstrating the role of spatial organization of TCR molecules in the control of TCR triggering (7–9), and it is also supported by our data showing significant differences in the number and size of TCR nanoclusters on naive CD4^+^ T cells depending on Flot2 expression. Further investigation is required to elucidate the precise mechanism of how the increased number of smaller TCR nanoclusters sensitizes the activation threshold. Additionally, understanding the effect of Flot2 on the formation of higher-order TCR clusters to create the T cell immunological synapse upon T cell activation will be of great interest.

We observed differential RNA splicing in Flot2-deficient CD4^+^ T cells in the naive, priming, and activated states, in particular, involving genes that regulate cell proliferation, activation, and protein translation (i.e., ribosomal protein-encoding genes). Taken together with our finding of increased levels of activation/maturation markers in lymph node CD4^+^ T cells of naive *Flot2^CD4^* mice (e.g., Nur77, CD44, T-bet, LFA-1α; Supplemental Figure 3), this may suggest that increased homeostatic TCR signaling in Flot2-deficient T cells primes alternate isoform usage in genes that support activation and proliferation upon subsequent TCR engagement. In support of the possibility that alternative RNA splicing can regulate TCR signal strength, it was recently reported that the splicing factor SRSF1 regulates T cell activation (67) and that alternative splicing of the adaptor protein MALT1 in response to TCR engagement controls CD4^+^ T cell activation (68).

Immune checkpoint blockade, a cornerstone of anticancer therapy, aims to inhibit T cell inhibitory receptors like PD-1 and CTLA-4 (69). However, this approach can lead to side effects such as T cell functional exhaustion and immune-related adverse events due to T cell overactivation (70–72). Our findings reveal that Flot2 deficiency enhances T cell responsiveness to low TCR stimulation, resulting in improved effector responses and tumor control in an in vivo tumor model, while mitigating T cell functional exhaustion. Importantly, neither global nor T cell–specific Flot2-KO mice exhibited the spontaneous autoimmune phenotypes seen in PD-1 or CTLA-4–KO mice (73–78), suggesting that therapeutic Flot2 targeting may be less subject to deleterious immunological side effects. Given its unique mechanism of regulating TCR nanocluster formation, Flot2 deficiency may hold promise for providing synergy in combination therapies with existing anticancer T cell approaches. Of interest, the more robust enhancement of antitumor immunity in the global Flot2-null mouse as compared with mice with T cell–specific Flot2 deletion (Figure 1A and Figure 2A) also raises the possibility that Flot2 deletion in non–T cells (e.g., DCs, fibroblasts, endothelial cells) may contribute to tumor suppression.

In conclusion, the present study demonstrates that Flot2 deletion can boost T cell antigen sensitivity as well as T cell effector functionality, potentially through regulating surface TCR clustering. These findings suggest that targeting Flot2 either in vivo or through engineering of T cells for adoptive cell therapy may hold promise for enhancing T cell reactivity in diseases with weak antigenicity, including cancer and chronic infections. We also posit that our findings suggest future avenues for investigating membrane-level mechanisms of receptor nanoclustering, for understanding differences in TCR signal transduction between CD4^+^ and CD8^+^ T cells, and potentially for how these differences may be leveraged therapeutically.

## Methods

### Sex as a biological variable.

Our studies were conducted using mice of both sexes, with sex-matched controls. Similar results were observed in both sexes.

### Mice.

*Flot2^fl/fl^* mice were generated by insertion of loxP sites flanking coiled-coil domain (14, 79) of the *Flot2* locus using standard cloning and homologous recombination methods involving electroporation of the targeting vector into mouse embryonic stem (ES) cells, followed by ES screening, blastocyst injection of appropriately targeted ES cells, and then breeding of male chimeras with WT C57BL/6 females. Subsequent global *Flot2* deletion was achieved by crossbreeding with CMV-Cre mice (The Jackson Laboratory, 006054), and T cell–specific deletion was achieved by crossbreeding with *CD4^cre^* transgenic mice (The Jackson Laboratory, 022071). All experiments used sex-matched controls approximately 6–12 weeks of age.

### Reagents.

For the flow cytometric analysis, following reagents were used: CellTrace Violet Cell Proliferation Kit (Invitrogen, C34557A), 7AAD (MilliporeSigma, A9400-1MG), anti-CD45.2 (BD Biosciences, 612778), anti-CD45 (Invitrogen, 48-0451-82), anti-TCRβ (BioLegend, 109246; BD Biosciences, 553170), anti-CD4 (BioLegend, 100549), anti-CD8α (BioLegend, 100741), anti-CD62L (eBioscience, 12-0621-81; Cytek, 60-0621-U025; BioLegend, 104436), anti-CD44 (BioLegend, 103047 or 740215), anti-CD69 (BioLegend, 104512), anti-CD25 (BioLegend, 102017; eBioscience, 47-0251-82), anti-Nur77 (Invitrogen, 12-5965-82), anti-Ki67 (BioLegend, 652413, 652403, or 652411), anti-LFA-1 (BioLegend, 141012), anti–T-bet (BioLegend, 644832; eBioscience, 53-5825-82), anti-TCF1 (Cell Signaling Technology, 6709S or 90511S), anti-TOX (Cell Signaling Technology, 44682S), anti-CXCR5 (BioLegend, 145522), anti–TIM-3 (BioLegend, 119723), anti–PD-1 (BD Biosciences, 744544; BioLegend, 135220), anti-Foxp3 (eBioscience, 12-4771-82; BioLegend, 126406), anti–TNF-α (BioLegend, 506308), anti–IFN-γ (BioLegend, 505826), anti–IL-2 (BD Biosciences, 557725), anti–IL-17 (BD Biosciences, 559502), anti–IL-10 (BioLegend, 505016), and anti–Granzyme B (BioLegend, 372204). For Western blots, the following reagents were used: RIPA Buffer (10×) (Cell Signaling Technology, 9806), Pierce RIPA Buffer (Thermo Fisher Scientific, 89901), NuPAGE LDS sample buffer (Invitrogen, NP0007), NuPAGE Bis-Tris Mini Protein Gels 4%–12% (Invitrogen, NP0335BOX), 4%–12% Criterion XT Bis-Tris Protein Gel (Bio-Rad, 3450125), NuPAGE MOPS SDS Running Buffer (Invitrogen, NP0001), Precision Plus Protein Kaleidoscope Prestained Protein Standards (Bio-Rad, 1610375), iBlot 2 Transfer Stacks (Invitrogen, IB24002), anti–phospho-Lck (Tyr505) (Cell Signaling Technology, 2751), anti–phospho–ZAP-70 (Cell Signaling Technology, 2717), anti–phospho-ERK1/2 (Cell Signaling Technology, 9101), anti-Lck (Cell Signaling Technology, 2752), anti–ZAP-70 (BD Biosciences, 610239), anti-ERK1/2 (Cell Signaling Technology, 9102), anti-Flot2 (Invitrogen, PA5-21296), goat anti–rabbit IgG (H+L) poly-hrp secondary antibody HRP (Invitrogen, 32260), anti–mouse IgG HRP-linked antibody (Cell Signaling Technology, 7076), anti–β-actin−peroxidase (Sigma-Aldrich, A3854), WesternBright Sirius (Advansta, K-12043-D10), Clarity Western ECL Substrate (Bio-Rad, 1705061), and Restore Western Blot Stripping Buffer (Thermo Fisher Scientific, 21059). For scRNA-Seq sample labeling, the following antibodies were used: TotalSeq-A0301 anti–mouse hashtag 1 (BioLegend, 155801), TotalSeq-A0302 anti–mouse hashtag 2 (BioLegend, 155803), and TotalSeq-A0303 anti–mouse hashtag 3 (BioLegend, 155805).

### FACS.

For the analysis of surface markers, cells were stained in PBS (Thermo Fisher Scientific) containing 1% (w/v) BSA (MilliporeSigma) and 0.09% sodium azide for 15 minutes on ice. Intracellular staining was conducted using the Foxp3/transcription factor staining buffer set (eBioscience) or the fixation/permeabilization kit (BD Biosciences) according to the manufacturer’s instructions. CTV labeling was performed according to the manufacturer’s instructions (Invitrogen), with slight modifications. Briefly, harvested cells were washed once with sterile PBS, and 1 × 10^6^ cells in PBS were stained with CTV at a final concentration of 5 μM for 30 minutes at 37°C. The labeling was stopped by adding 10% FBS in PBS, followed by 1 wash, and the cells were resuspended for subsequent analysis. To assess cytokine production by FACS, cells were stimulated with 50 ng/mL phorbol 12-myristate 13-acetate (PMA; MilliporeSigma), 0.5 μM ionomycin (MilliporeSigma), and 2 μM monensin (BioLegend) for 4 hours at 37°C.

### In vitro stimulation of naive T cells.

Naive CD4^+^ or CD8^+^ T cells were isolated from mouse spleen and lymph nodes by using a mouse naive CD4^+^ or CD8^+^ T cell isolation kit (Stemcell Technologies). Purified naive CD4^+^ or CD8^+^ T cells were labeled with CTV (Invitrogen) and cultured in RPMI-1640 (Thermo Fisher Scientific) complete medium supplemented with 10% FBS, 57.2 μM β-mercaptoethanol, and 1× antibiotic-antimycotic (Thermo Fisher Scientific) at a density of 1 × 10^5^ cells per well in a 96-well flat-bottom plate. Naive CD4^+^ or CD8^+^ T cells were stimulated with various concentrations of plate-bound αCD3 (BioLegend, 100253) and a fixed concentration of soluble αCD28 (BioLegend, 102121; 1 μg/mL) antibodies for different time points as indicated in the figure legends.

### In vitro polarization of Th cells.

Naive CD4^+^ T cells were purified from mouse spleen and lymph nodes by using a mouse naive CD4^+^ T cell isolation kit (Stemcell Technologies) and polarized following the manufacturer’s protocol for Immunocult Mouse Th1 or Th2 differentiation supplements (Stemcell Technologies). For Th17 polarization, naive CD4^+^ T cells were cultured with 5 ng/mL TGF-β and 20 ng/mL IL-6, while Treg polarization was induced by culturing cells with 5 ng/mL rhIL-2 and 5 ng/mL TGF-β, both for 3.5 days. All Th cell polarization involved cell stimulation using the indicated concentration of plate-bound αCD3 and 0.5 μg/mL soluble αCD28. Polarization efficiency was assessed by quantifying cytokine production and lineage marker expression via FACS.

### scRNA-Seq and data processing.

Single-cell suspensions of stimulated *Flot2^WT^* or *Flot2^CD4^* naive CD4^+^ T cells were washed, loaded, and processed for 10X Genomics scRNA-Seq analysis. Fastq files were processed using Cell Ranger’s “multi” functionality for hashtag oligos using information of the 2 separate sequencing outputs, Cell Ranger’s mm10-3.0.0 reference, and associated oligo hashtag information. Data were analyzed using Seurat v5.0.1 (80) in R4.3.1 as previously (81). In short, the scRNA-Seq dataset from 2 sample lanes was merged with hashtag information (3 antibodies per sample lane). Hashtag information was demultiplexed and classified using the “HTODemux” function, and subset only on cells that were assigned singlet (i.e., containing one hashtag) classification, to generate 6 separate samples in total. Samples were then filtered for homogeneity, using the following parameters: nFeature_RNA (500–4,250 features), nCount_RNA (100–20,000 counts), percent mitochondria (0.0075%–0.0800%), percent cycling (0.0075%–0.0800%). Count matrices were normalized and scaled for number of RNA features, proportion cycling, and proportion mitochondrial content. Subsequently, data dimensions were reduced by PCA and by UMAP (using the first 30 PCs). Clustering was performed using the “FindClusters” function using the Louvain algorithm (k.param = 50, resolution = 0.5). Genes identifying cluster membership were generated by the “FindAllMarkers” function. Spliced and unspliced count matrices were generated with the “velocyto run” function using the same reference genes as Cell Ranger and the same bam files generated from Cell Ranger (82). RNA velocity results were displayed using velocyto.R v0.6 (82).

### Super-resolution imaging and analysis of TCR nanoclusters.

To visualize TCR distribution in the plasma membrane, staining was conducted as previously described with a modification (57). Briefly, 5 × 10^5^ *Flot2^WT^* or *Flot2^CD4^* naive CD4^+^ T cells were fixed with 4% PFA, followed by surface staining with 5 μg/mL anti-mCD3ε (Invitrogen, MA1-10184) for 4 hours at 4°C. Subsequently, the cells were stained with 2 μg/mL Alexa Fluor 647–conjugated goat anti–hamster IgG (BioLegend, 405510) for 2 hours at 4°C after washing with PBS 10 times. In cases where Alexa Fluor 647–conjugated anti-mTCRβ antibody (BioLegend, 109218) was used, secondary staining was omitted. Stained cells were resuspended in 40 μL PBS and transferred to the poly-D-lysine coated dish, followed by overnight incubation at 37°C for cell attachment to the dish bottom. A mercaptoethylamine-based STORM cocktail was used for super resolution imaging through localization microscopy. Three solutions were made consisting of: Solution A (0.8mL) – 30mM Tris/Cl pH 8.5 containing 1 mM EDTA and 6.25 μM glucose oxidate + 2.5 μM catalase, Solution B (0.1mL) – 250 mM cysteamine-HCL in water, and Solution C (0.1mL) – 250 mM glucose in water. Solution A was gently mixed with Solution B, and this combined mixture was gently added to Solution C. This ABC mixture was immediately pulled into a gas-tight glass syringe with a PEEK needle minimizing any air bubbles. The PBS solution from the fixed/labeled cells attached to the poly-D-lysine–coated dish was removed, and a 25 mm square coverslip was placed over the 14 mm microwell. Then, the PEEK needle from the glass syringe was used to inject the ABC solution across the sample to deplete dissolved oxygen from the sample chamber. In total, 10,000 images were captured in burst mode on an Andor Dragonfly 505 imaging system using its 3D astigmatic lens for 3D super-resolution imaging. A 637 nm laser at 100% with the PD4 power density setting was used to excite Alexa647 through a Nikon CFI Aprochromat TIRF 60× Oil Immersion lens and the corresponding fluorescence emission was captured through a 660–738 nm emission filter and Andor iXon EMCCD camera with an exposure of 10 msec. This image series was then taken into Huygens Localizer (v22.10, Scientific Volume Imaging) for generation of a 3D localization table using the Weighted Least-squares fit method with a 3D Z-position calibration point spread function (PSF) and drift correction applied. The 3D localization table was then opened with Huygens Cluster Analyzer 22.10 or 23.10, where the FOCAL algorithm (nearestNeighborsCross 7, threshold 20, and minimal voxel count 9) or the DBSCAN algorithm (a minimal neighbors 6 and minimal cluster size 1) were used to identify CD3ε or TCRβ clusters, respectively.

### L. monocytogenes infection.

Mice were infected with *L*. *monocytogenes* via retro-orbital i.v. injection, receiving a dosage of 5,000 colony-forming units (CFU) per mouse. Daily monitoring of weight loss after infection was conducted and subsequently analyzed. Spleens were excised and mechanically dissociated to obtain total splenocytes. Cytokine production and the expression of surface and intracellular markers were analyzed by FACS.

### Tumor models.

In total, 5 × 10^5^ B16F10 or MC38 cells were injected intradermally into *Flot2^+/+^* and *Flot2^–/–^* mice and s.c. into *Flot2^WT^* and *Flot2^CD4^* mice. Tumor size was monitored every 2–3 days starting from day 5 or day 7. Tumor size was calculated as: (length × width^2^)/2. Tumor-bearing mice were monitored and analyzed until day 20. The experimental endpoint was determined based on tumor size (with a limit of 2,000 mm^3^), severe tumor ulcerations, or other health issues that conflicted with the approved animal study protocol by the Animal Care and Use Committee of the NIEHS. Tumors and dLNs were excised and mechanically dissociated, and T cells in each tissue were analyzed by FACS.

### ELISPOT assay.

Splenocytes from B16F10 tumor–bearing mice were stimulated with 1 μg/mL TRP-2 melanoma peptide (SVYDFFVWL) for 24 hours, followed by an IFN-γ ELISPOT assay to measure antigen-specific reactivity.

### Mixed bone marrow chimera.

Mixed bone marrow chimera experiments were performed as previously described (34). Briefly, bone marrow from either WT or *Flot2^–/–^* mice were mixed with bone marrow from *TCR*α*^–/–^* mice at a 1:5 ratio and transferred into *TCR*α*^–/–^* recipient mice that were lethally irradiated using 1,100 rads. After 10 weeks of reconstitution, 2 × 10^5^ B16F10 tumor cells were intradermally injected, and tumor growth and TILs were analyzed.

### Quantitative PCR.

Total RNA was obtained using RNeasy kits (Qiagen) following manufacturer instructions. cDNA was synthesized using the iScript cDNA synthesis kit (Bio-Rad). cDNA was quantified with TaqMan Universal PCR Master Mix (Invitrogen) and predesigned TaqMan primers (Assay ID: Mm00514962_g1, Mm01241315_g1; both Flot2). The ΔΔCt method was utilized for analyzing the fold change in gene expression, which was normalized using appropriate reference genes. QuantStudio Software (Thermo Fisher Scientific) was used for data analysis.

### Western blot.

To measure phosphorylation of TCR signaling molecules, naive CD4^+^ T cells were isolated from mouse spleen and lymph nodes using a mouse naive CD4^+^ T cell isolation kit (Stemcell Technologies). The cells were then rested in plain RPMI media at 37°C for 2 hours prior to stimulation. Rested naive CD4^+^ T cells were stimulated for 3 minutes at a density of 2 × 10^5^ cells per well in a 96-well plate precoated with varying concentrations of plate-bound αCD3, as specified in the figures. Stimulation was terminated by either adding 10× RIPA buffer, removing the media by pipetting and immediately adding 1× RIPA buffer, or adding 4× LDS with 20× dithiothreitol (DTT; 1M). For samples treated with 10× or 1× RIPA buffer, cells were lysed on ice for 30 minutes, followed by centrifugation at 18,800*g* for 15 minutes at 4°C to remove the insoluble fraction. The supernatant was collected, mixed with 4× LDS and 20× DTT, and boiled for 10 minutes at 70°C. For samples where 4× LDS with 20× DTT was directly added, lysates were boiled for 10 minutes at 95°C. Western blotting was performed using an equal number of cells and equal volumes of lysed samples, normalized to the respective total protein levels. To measure Flot2 expression in tissues, the tissues of interest were dissected and snap-frozen by immersion in liquid nitrogen. The frozen tissues were then mechanically homogenized in cold 1× RIPA buffer using a bead homogenizer. The homogenates were agitated for 2 hours at 4°C, transferred to new tubes, and centrifuged at 16,000*g* for 20 minutes at 4°C. The supernatant was collected for a BCA assay. Samples were then mixed with 4× LDS and 20× DTT and boiled for 10 minutes at 95°C, and equal amounts of protein were used for Western blotting.

### Statistics.

Statistical analyses were conducted using GraphPad Prism (GraphPad Software Inc.). Comparisons between 2 groups were calculated using unpaired 2-tailed Student’s *t* tests, and multiple comparisons were performed using 1-way or 2-way ANOVA with Šidák’s multiple-comparison tests. A χ^2^ analysis and the Wilcoxon rank-sum test were used for genotype occupancy analysis and spliced RNA analysis in scRNA-Seq, respectively. A *P* value less than 0.05 was considered significant.

### Study approval.

All experiments were performed in accordance with the Animal Welfare Act and the US Public Health Service Policy on Humane Care and Use of Laboratory Animals after review by the Animal Care and Use Committee of the NIEHS.

### Data availability.

The authors confirm that the data associated with the manuscript and supplemental material are provided in the Supporting Data Values file. scRNA-Seq expression data are available in the NCBI Gene Expression Omnibus repository (accession no. GSE275696).

## Author contributions

SM conceptualized the study, conducted experiments, analyzed the data, and drafted the initial manuscript. FZ also conceived the study, conducted experiments, and participated in data analysis. MNU conducted Western blot experiments, while CJT aided in data acquisition and analysis of super-resolution imaging. PWFK analyzed scRNA-Seq data and offered intellectual input on experimental design and analysis in general. MBF supervised the project, contributed to experimental design and analysis, and provided overall guidance. All authors contributed to manuscript drafting.

## Supplementary Material

Supplemental data

Unedited blot and gel images

Supporting data values

## Version 1 11/05/2024

In-Press Preview

## Version 2 12/20/2024

Electronic publication
