# Supplementary material for: Flotillin-2 dampens T cell antigen sensitivity and functionality
Source: JCI Insight. 2024 Dec 20;9(24):e182328. doi: 10.1172/jci.insight.182328 (PMC11665568; doi:10.1172/jci.insight.182328)
Supplement: Supplemental data [file jciinsight-9-182328-s008.pdf]

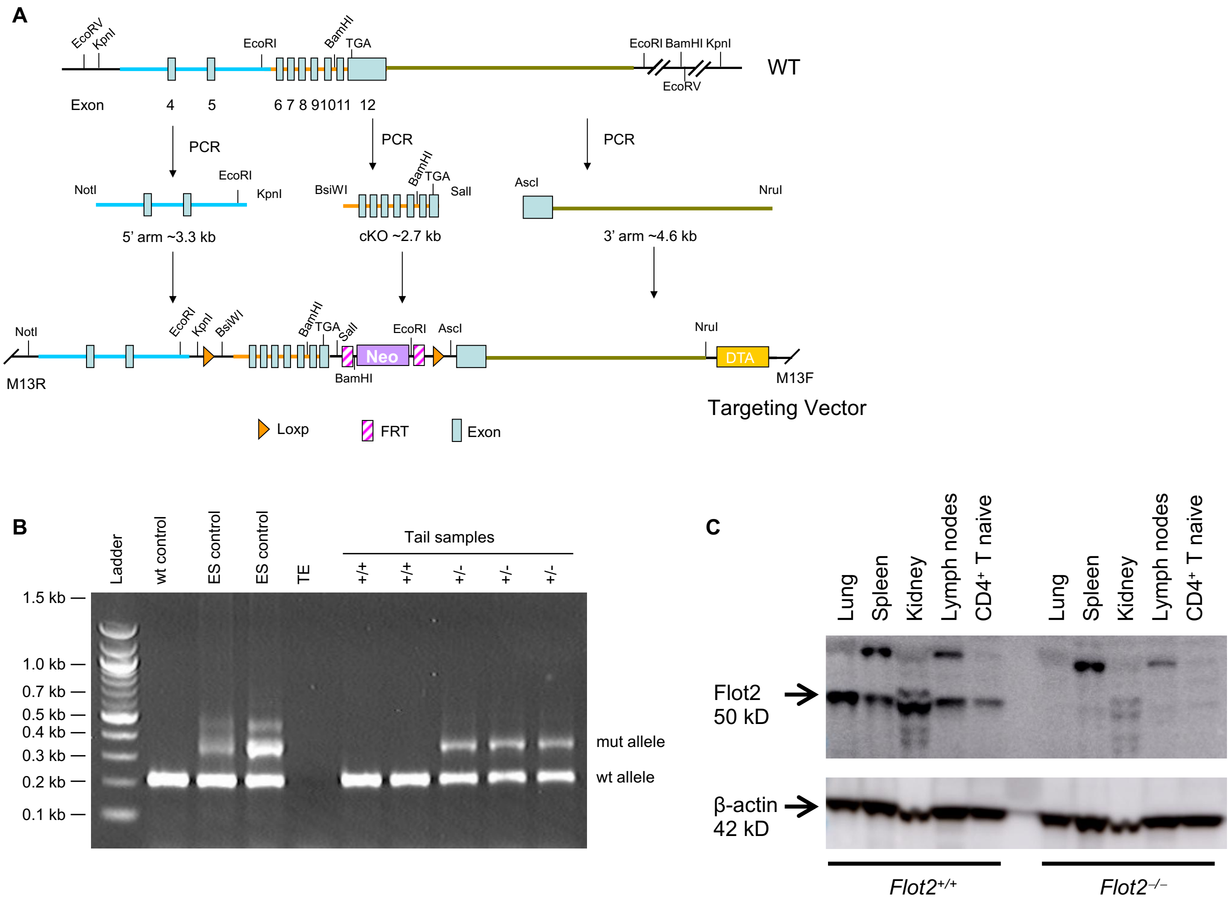

Supplemental Figure 1. **Flotillin knockout mouse targeting vector strategy and confirmation.**
(**A**) The vector targeting strategy for flanking the murine *Flot2* locus with loxP sites is shown, along with restriction sites. (**B**) Pups from chimera breeding were screened by PCR of tail samples using the following primers: 5'-ATCACTGTCTGTCTGTGAGGAGTGG-3' and 5'-AGGGCAAGAGCGTGTGGGTTGTGG-3' followed by gel electrophoresis as shown. (**C**) Loss of Flot2 expression at the protein level was confirmed in *Flot2*$^{-/-}$ mice using Western blot analysis.

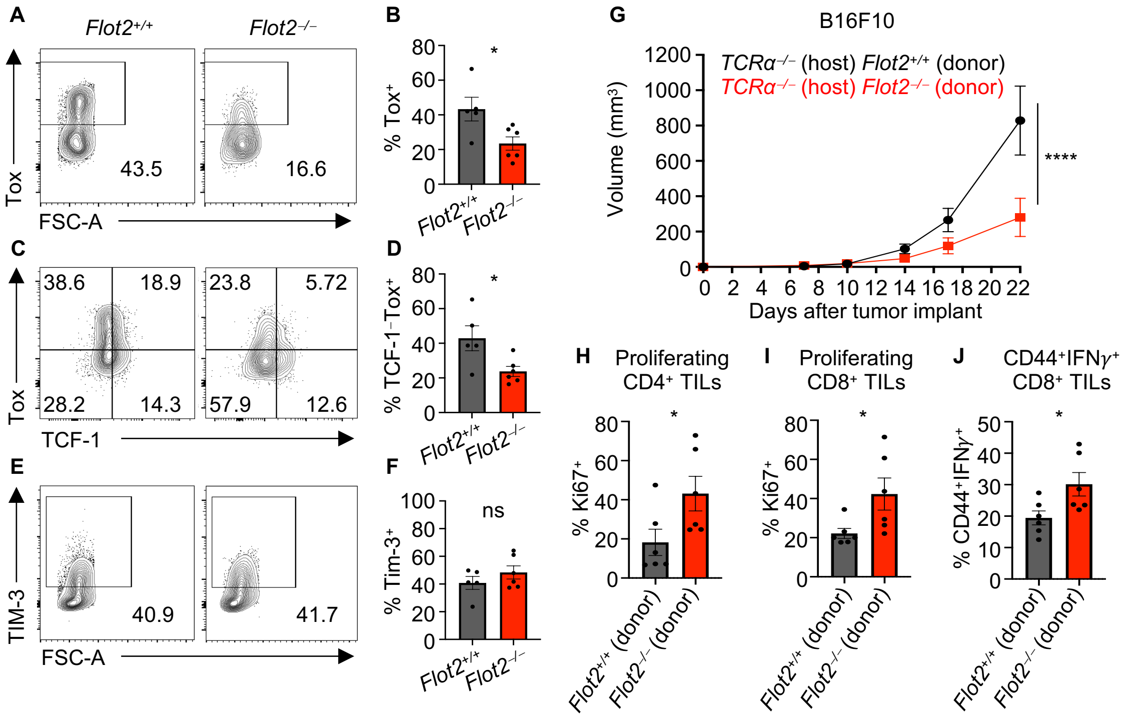

Supplemental Figure 2. Flot2 deficiency reduces T cell functional exhaustion and enhances anti-tumor T cell responses. (**A**-**F**) Flow cytometric analysis of TILs in MC38 tumor-bearing *Flot2*$^{+/+}$ or *Flot2*$^{-/-}$ mice: Representative plots (**A**, **C**, and **E**) are shown. Tox^{+} (**B**), TCF-1^{-}Tox^{+} (**D**), and Tim-3^{+} (**F**) populations within 7AAD^{-}CD45.2^{+}TCRβ^{+}CD8^{+} population are depicted. (**G**) B16F10 tumor volume in mixed bone marrow chimeras reconstituted with a 1:5 ratio mixture of bone marrow cells from either *Flot2*$^{+/+}$ or *Flot2*$^{-/-}$ mice and *TCRα*$^{-/-}$ mice (n = 13 per group). (**H**-**J**) Flow cytometric analysis of TILs. Ki67^{+} populations among 7AAD^{-}CD45^{+}CD4^{+} population (**H**) or 7AAD^{-}CD45^{+}CD8^{+} population (**I**), and CD44^{+}IFNγ^{+} populations within 7AAD^{-}CD45^{+}CD8^{+} population (**J**) are shown. Data are representative of two independent experiments (**A**-**F** and **H**-**J**) or pooled from two independent experiments (**G**). Data were analyzed by unpaired t-test (**B**, **D**, **F**, and **H**-**J**) or two-way ANOVA (**G**). Error bars denote SEM; *P<0.05; ****P<0.0001. ns = non-significant.

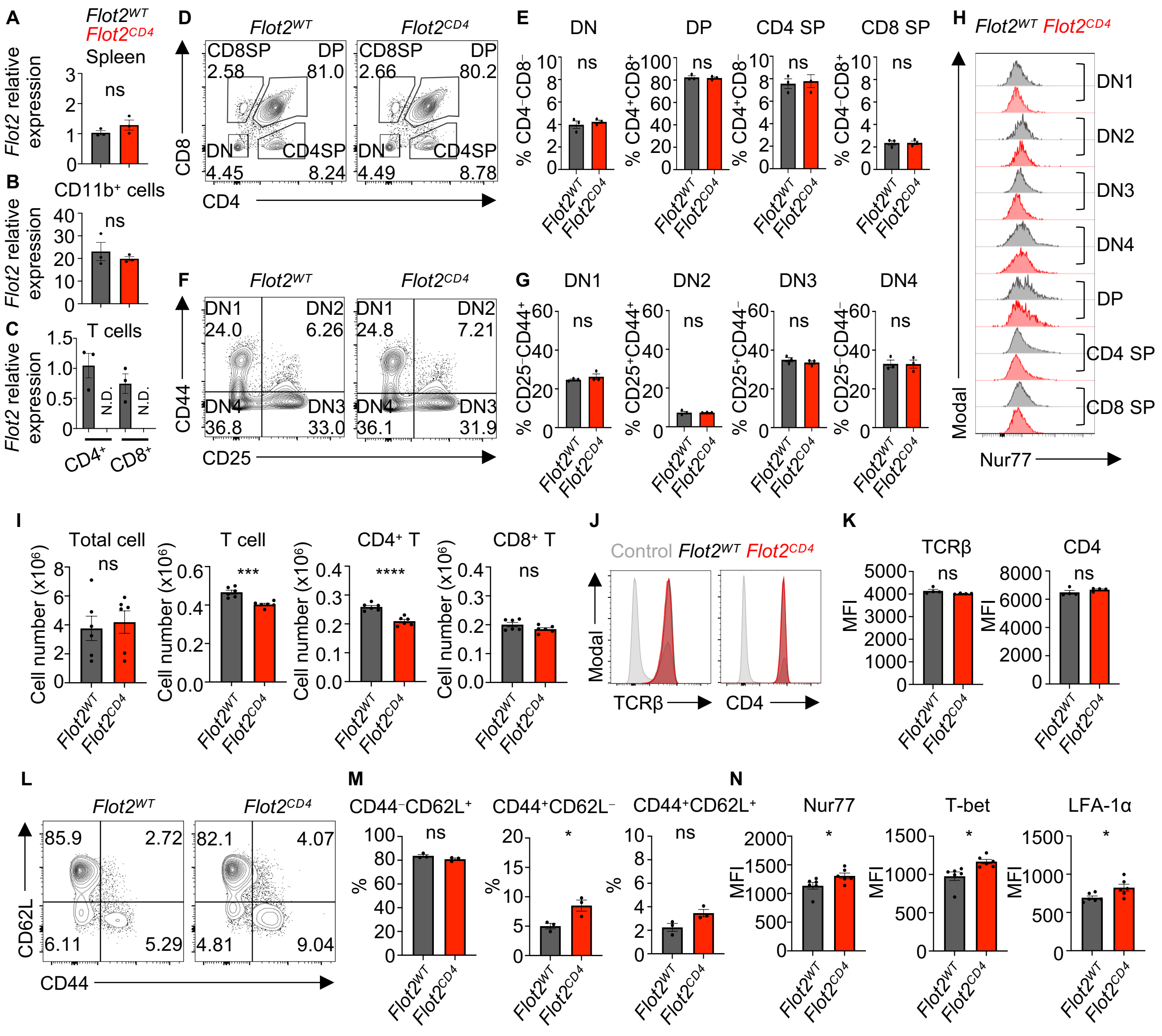

Supplemental Figure 3. Steady state analysis of *Flot2*^{WT} and *Flot2*^{CD4}.

(**A**-**C**) Flot2 mRNA expression was comparable in total spleen (**A**) or CD11b^{+} cells (**B**) but was fully deleted in both CD4^{+} and CD8^{+} T cells of *Flot2*^{CD4} mice (**C**). (**D**-**H**) At steady state, the thymus tissues from *Flot2*^{WT} and *Flot2*^{CD4} mice were removed, and thymocytes were isolated using mechanical dissociation, followed by FACS analysis. Representative plots (**D** and **F**) are shown. CD4^{-}CD8^{-} (DN), CD4^{+}CD8^{+} (DP), CD4^{+}CD8^{-} (CD4 SP), and CD4^{-}CD8^{+} (CD8 SP) populations among singlet thymocytes (**E**) and CD25^{-}CD44^{+} (DN1), CD25^{+}CD44^{+} (DN2), CD25^{+}CD44^{-} (DN3), CD25^{-}CD44^{-} (DN4) populations within the DN subset (**G**) in steady state *Flot2*^{WT} or *Flot2*^{CD4} mice are presented. Flow cytometric histogram plot of Nur77 expression at each stage of thymocytes are displayed (**H**). (**I**) Cell numbers of total cells, T cells, CD4^{+} T cells, CD8^{+} T cells in inguinal lymph nodes of steady state *Flot2*^{WT} or *Flot2*^{CD4} mice. (**J** and **K**) Flow cytometric analysis of TCRβ and CD4 expression on viable lymphocytes at steady state. Representative plots (**J**) and mean fluorescence intensity (MFI) quantification (**K**) are shown. (**L**-**N**) Flow cytometric analysis of naïve, effector, central memory populations and Nur77, T-bet, LFA-1α expression within steady state lymph node CD4^{+} T cells. Representative plots of each populations are shown (**L**). CD44^{-}CD62L^{+} (naive), CD44^{+}CD62L^{-} (effector), CD44^{+}CD62L^{+} (central memory) populations (**M**) and MFI quantification of Nur77, T-bet, LFA-1α (**N**) are provided. Data are representative of two independent experiments (**A**-**N**). Data were analyzed by unpaired t-test (**A**-**C**, **E**, **G**, **I**, **K**, **M**, and **N**). Error bars denote SEM; *P<0.05; ***P<0.001; ****P<0.0001. ns = non-significant. N.D. = not detected.

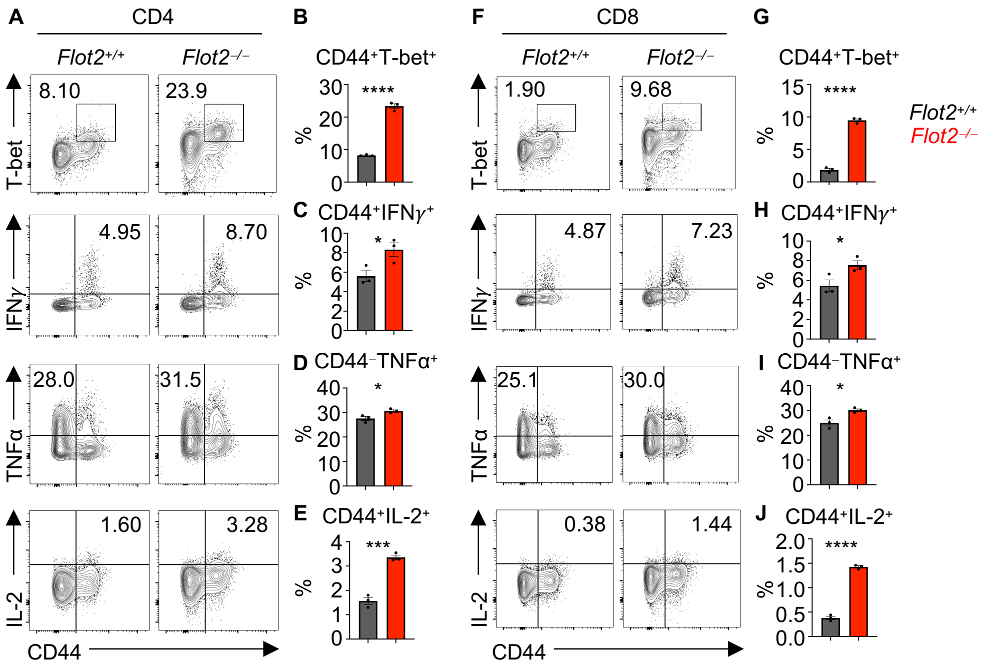

Supplemental Figure 4. Flot2 deletion boosts effector T cell responses in Listeria monocytogenes-infected mice. (**A**-**J**) Flow cytometric analysis of splenic T cells from *Listeria*-infected *Flot2*$^{+/+}$ or *Flot2*$^{-/-}$ mice: Representative plots (**A** and **F**) are shown. $CD44^{+}$T-bet^{+} (**B**), $CD44^{+}IFN\gamma^{+}$ (**C**), $CD44^{-}TNF\alpha^{+}$ (**D**), and $CD44^{+}$IL-2^{+} (**E**) populations within viable $CD45^{+}TCR\beta^{+}CD4^{+}$ population, and $CD44^{+}$T-bet^{+} (**G**), $CD44^{+}IFN\gamma^{+}$ (**H**), $CD44^{-}TNF\alpha^{+}$ (**I**), and $CD44^{+}$IL-2^{+} (**J**) populations within viable $CD45^{+}TCR\beta^{+}CD8^{+}$ population are shown. Data are representative of two independent experiments (**A**-**J**). Data were analyzed by unpaired t-test (**B**-**E** and **G**-**J**). Error bars denote SEM; *$P<0.05$; ***$P<0.001$; ****$P<0.0001$.

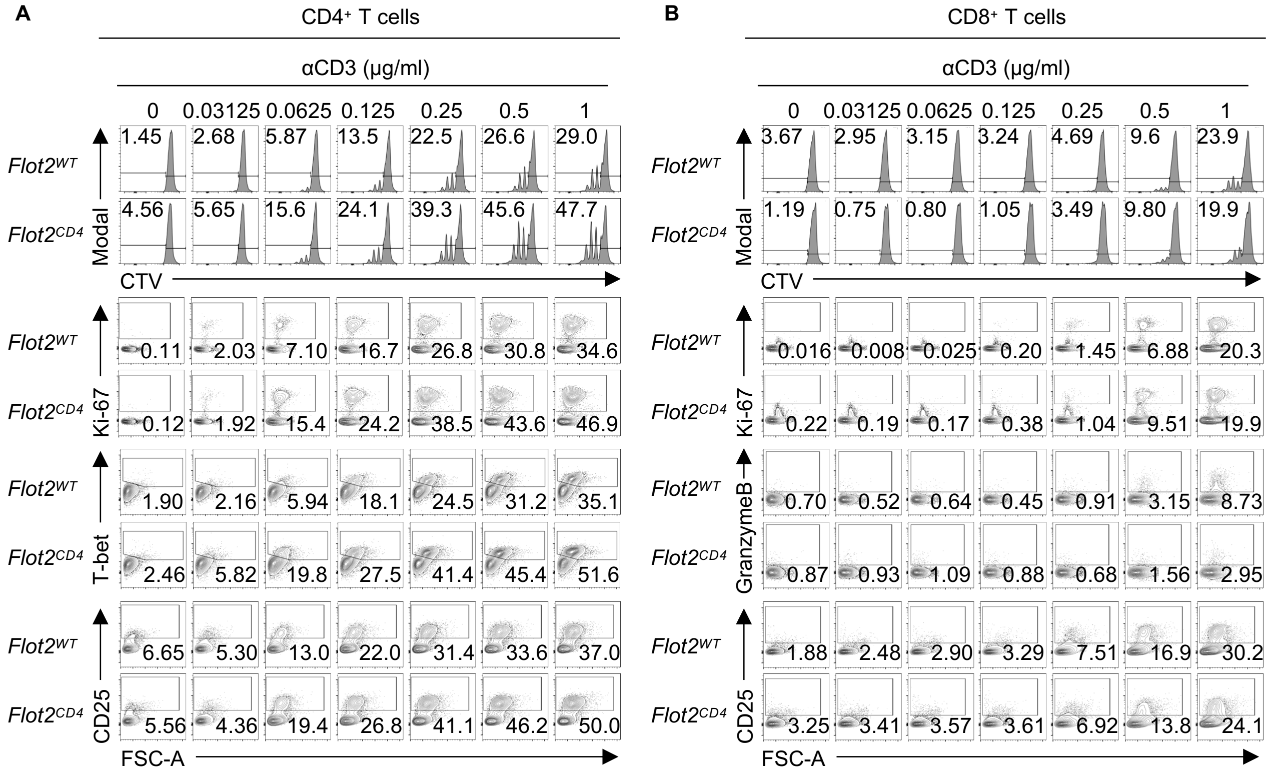

Supplemental Figure 5. *Flot2^{CD4}* CD4$^+$ but not CD8$^+$ T cells displayed enhanced responses to in vitro stimulation.
Representative plots of flow cytometric analysis of naïve CD4$^+$ (**A**) or CD8$^+$ T cells (**B**) that were stimulated in vitro for 72 hours with varying doses of plate-bound αCD3, alongside a fixed dose of soluble αCD28 (1 μg/ml). CTV$^-$, Ki67$^+$, T-bet$^+$, and CD25$^+$ populations within viable TCRβ$^+$CD4$^+$ population (**A**) or CTV$^-$, Ki67$^+$, Granzyme B$^+$, and CD25$^+$ populations within viable TCRβ$^+$CD8$^+$ population (**B**) are shown. Data are representative of two independent experiments (**A** and **B**).

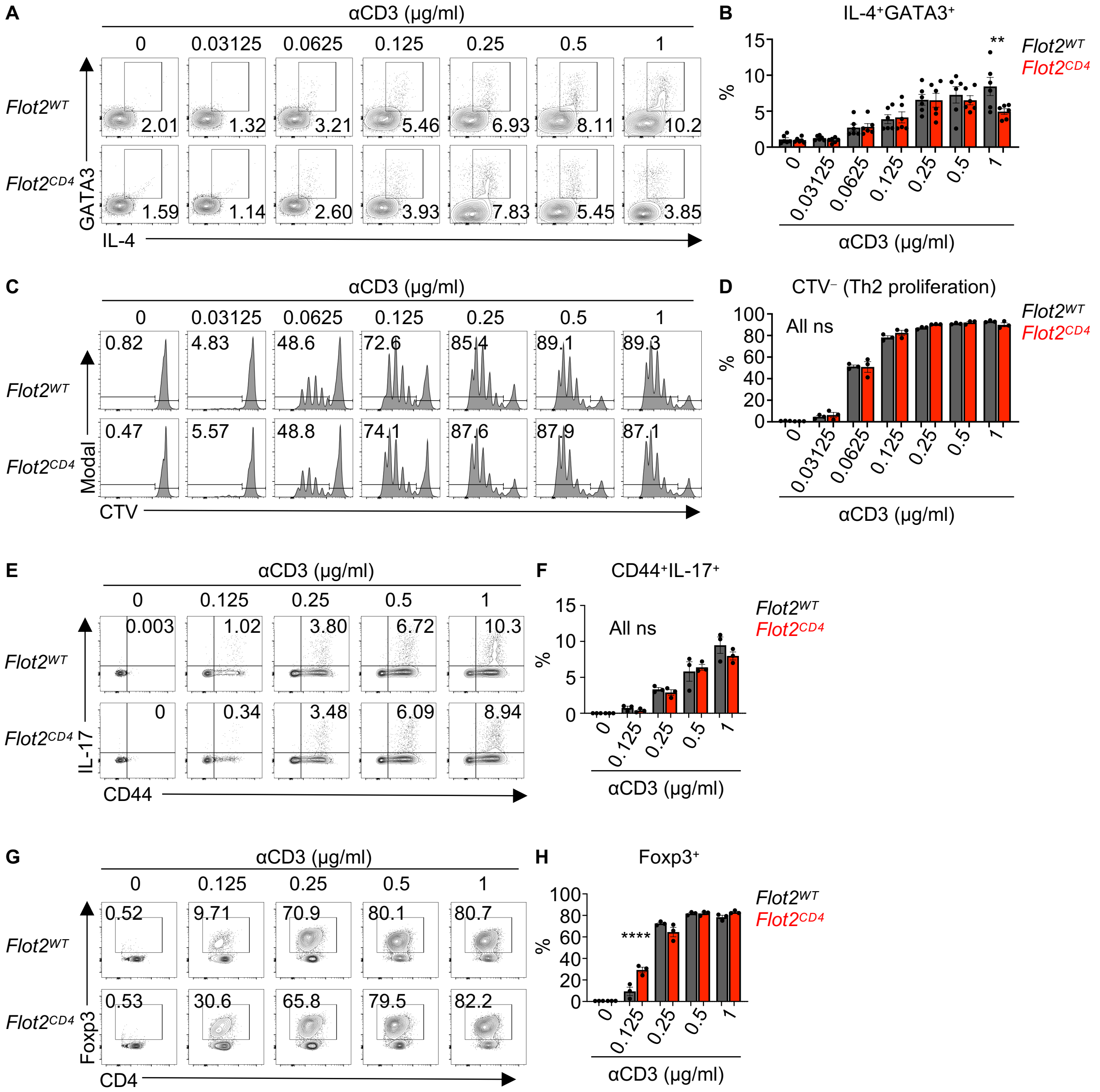

Supplemental Figure 6. Flot2 ablation does not affect Th2 and Th17 differentiation, while enhancing Treg differentiation at weak TCR stimulation.

(**A**-**H**) Naïve CD4+ T cells were purified and differentiated towards the Th2 (**A**-**D**), Th17 (**E** and **F**) and Treg (**G** and **H**) subtypes in vitro, followed by flow cytometric analysis. Representative plots (**A**, **C**, **E**, and **G**) are shown. IL-4+GATA3+ (**B**), CTV− (**D**), CD44+IL-17+ (**F**), and Foxp3+ (**H**) populations within viable TCRβ+CD4+ population are shown. Data are representative of two independent experiments (**A**-**H**). Data were analyzed by one-way ANOVA followed with Sidak's multiple comparison tests (**B**, **D**, **F**, and **H**). Error bars denote SEM; **P<0.01; ****P<0.0001. ns = non-significant.

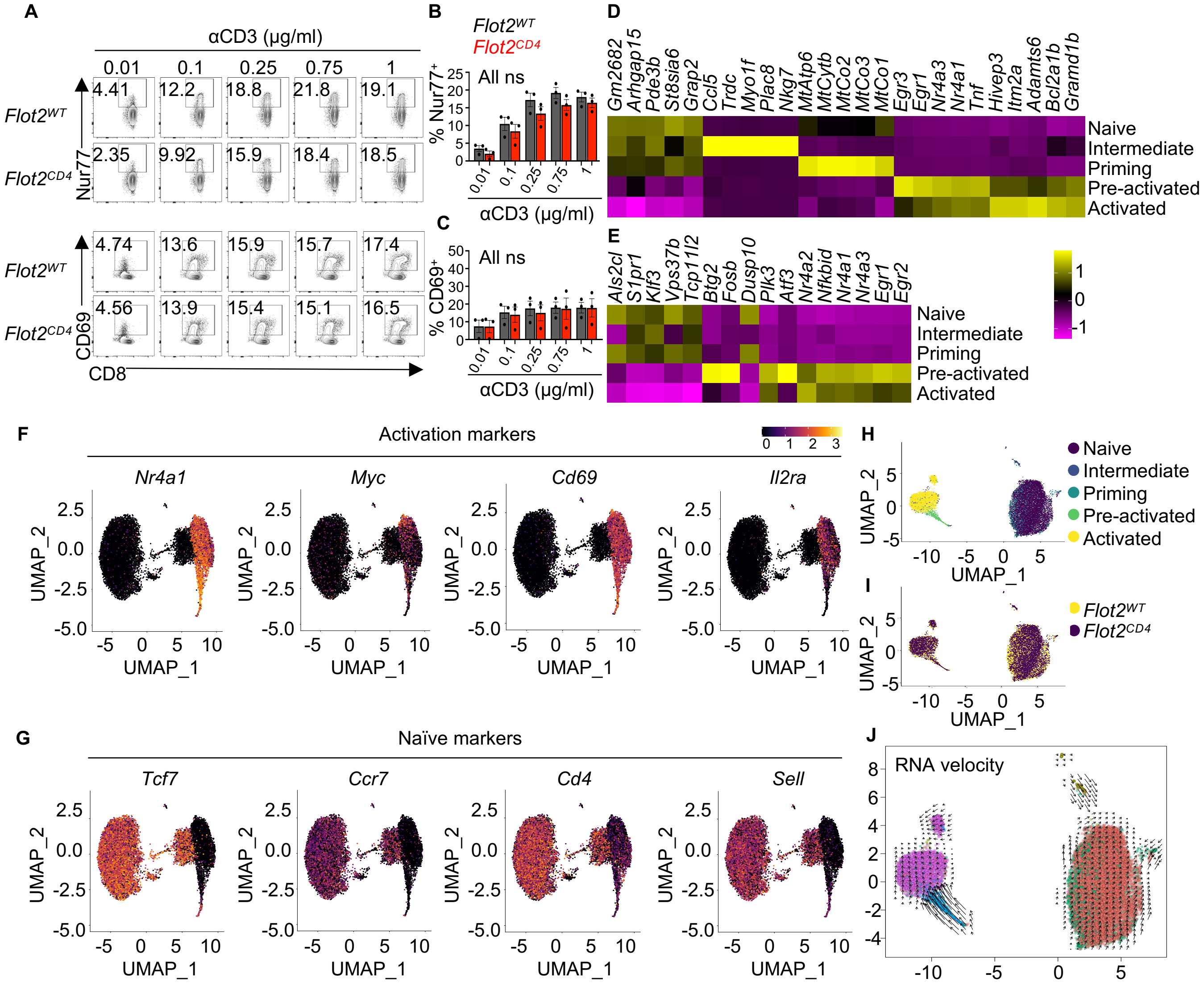

Supplemental Figure 7. Flot2 ablation does not impact the early activation of CD8⁺ T cells upon in vitro stimulation, nor does it affect the transcriptional activation trajectory of CD4⁺ T cells.
(**A**-**C**) Naïve CD8⁺ T cells were purified and stimulated in vitro for 3 hours (**B**) or 24 hours (**C**) with varying doses of plate-bound αCD3, alongside a fixed dose of soluble αCD28 (1 μg/ml), followed by flow cytometric analysis to assess TCR signaling (Nur77) and early T cell activation (CD69). Representative plots (**A**) are shown. Nur77⁺ (**B**) and CD69⁺ (**C**) populations within viable TCRβ⁺CD8⁺ population are indicated. (**D and E**) Gene expression heatmap from scRNA-seq analysis. Top markers for clustering (**D**) and gene set related to early T cell activation (**E**) are shown. (**F and G**) Expression of T cell activation (**F**) or naïve state (**G**) marker genes over the UMAP dot plots. (**H**-**J**) T cell activation trajectory fitted by RNA velocity analysis. T cell functional clustering (**H**), genotype (**I**), and RNA velocity (**J**) results are shown. Data are pooled from three independent experiments and were analyzed by one-way ANOVA followed with Sidak's multiple comparison tests (**A**-**C**). Error bars denote SEM. ns = non-significant.

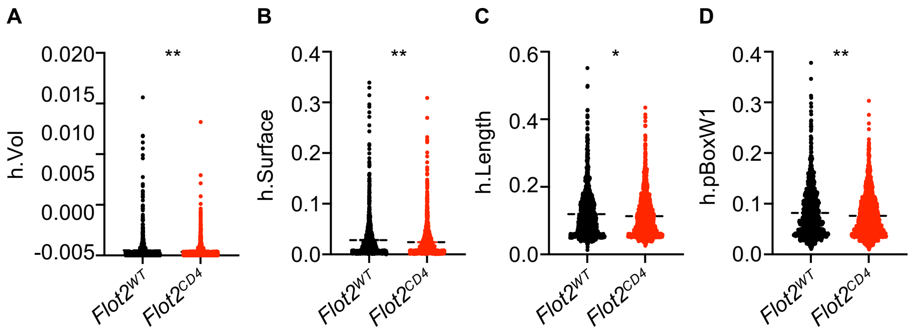

Supplemental Figure 8. Convex hull geometry analysis of TCR nanoclusters.
(**A**-**D**) Convex hull geometry analysis of TCRβ⁺ nanoclusters in *Flot2^{WT}* and *Flot2^{CD4}* naïve CD4⁺ T cells: Volume enclosed by the convex hull of the cluster in cubic micrometers (h.Vol; **A**), surface of the convex hull of the cluster in squared micrometers (h.Surface; **B**), the largest length of the convex hull of the cluster in micrometers (h.Length; **C**), and the largest width of the convex hull principal box perpendicular to the convex hull length in micrometers (h.pBoxW1; **D**) are quantified and displayed. Data are representative of two independent experiments (**A**-**D**). Data were analyzed by unpaired t-test (**A**-**D**). Error bars denote SEM; *P<0.05; **P<0.01.

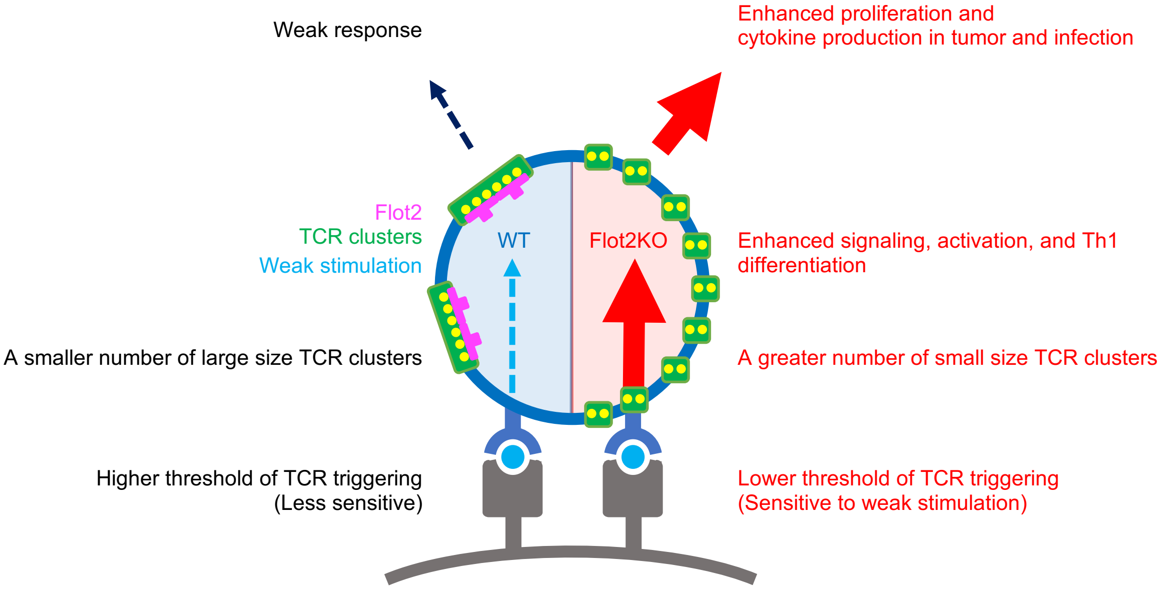

Supplemental Figure 9. Flotillin-2 dampens T cell antigen-sensitivity and functionality.
