# Supplementary material for: Flotillin-2 dampens T cell antigen sensitivity and functionality
Source: JCI Insight. 2024 Dec 20;9(24):e182328. doi: 10.1172/jci.insight.182328 (PMC11665568; doi:10.1172/jci.insight.182328)

Western Blot original files

Full unedited gels for Supplementary Figure 1C

Flot2

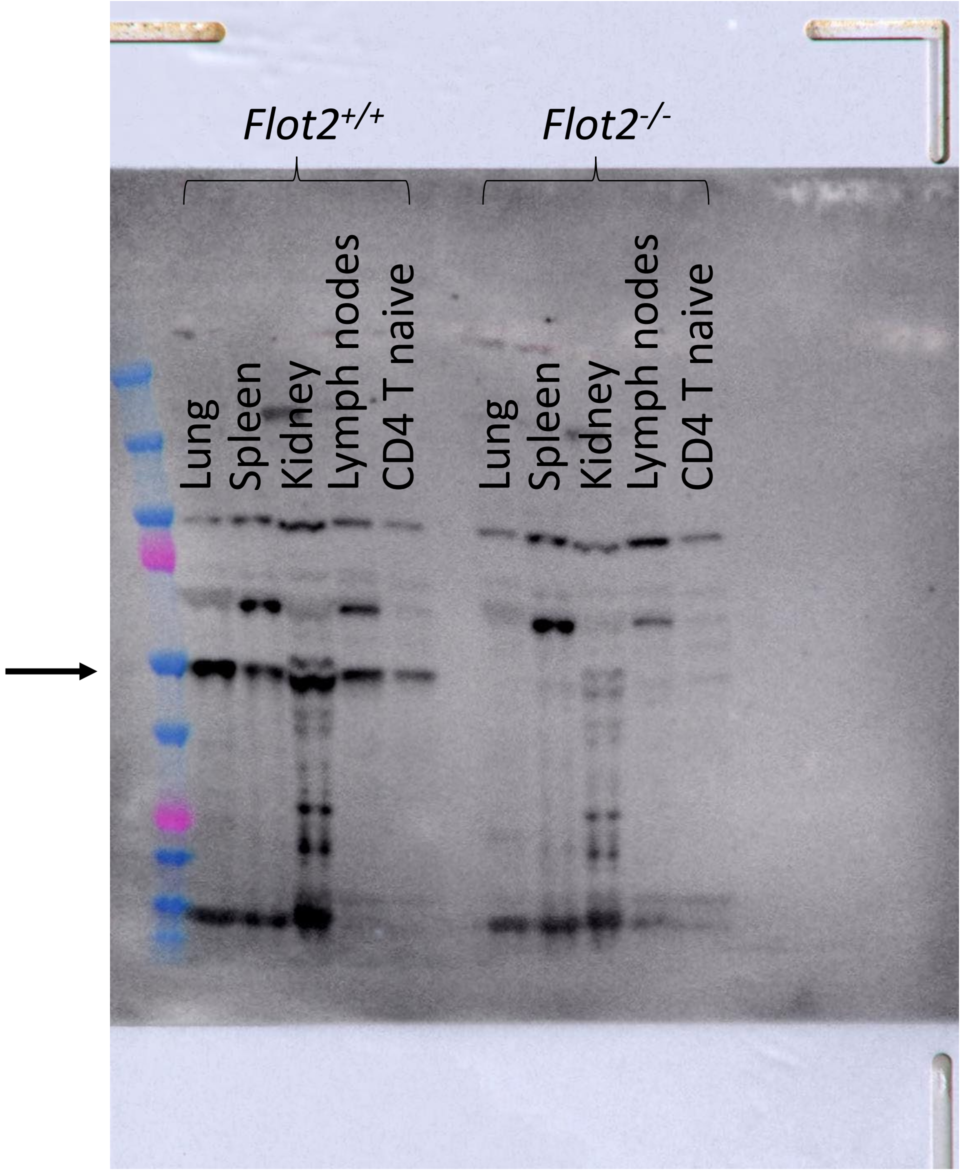

Beta-actin

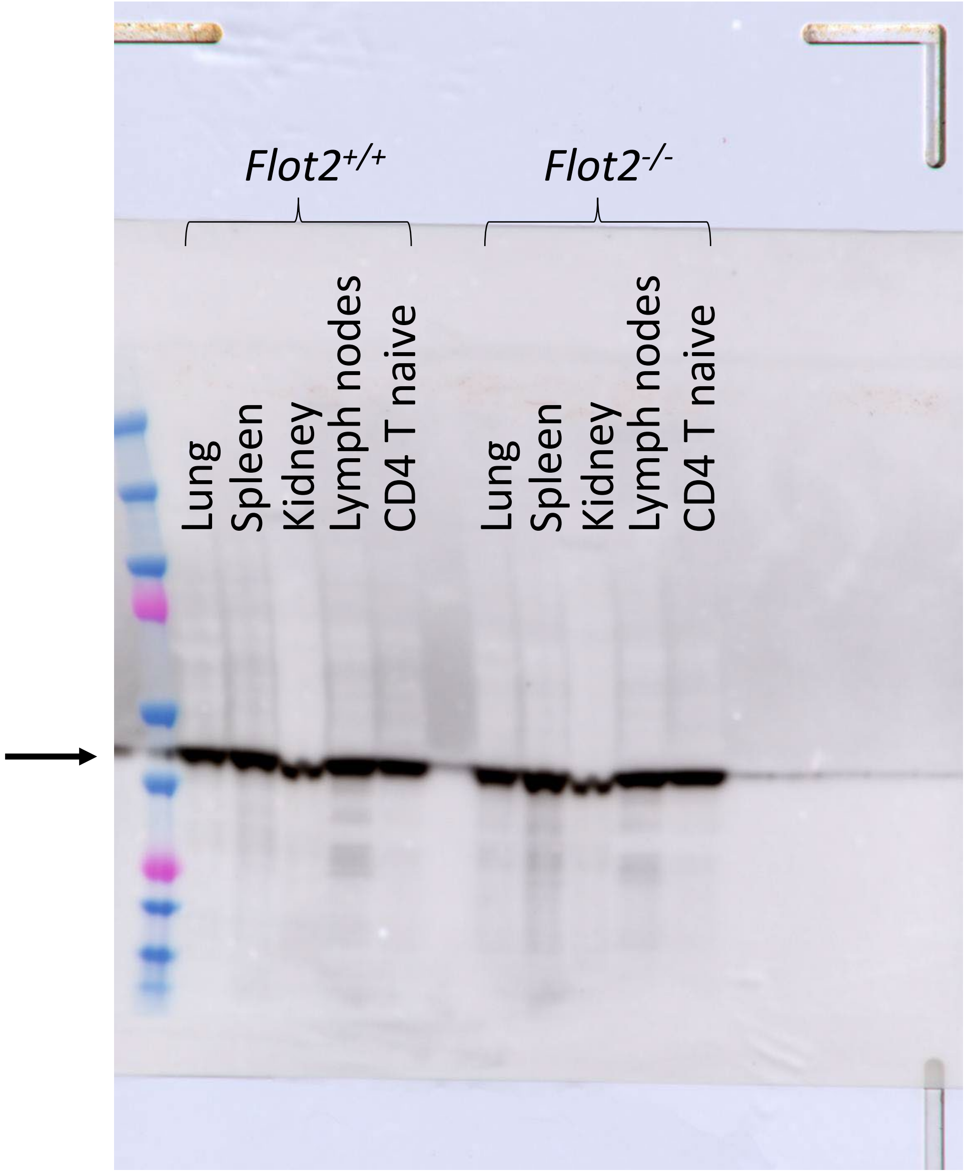

Full unedited gels for Figure 6D

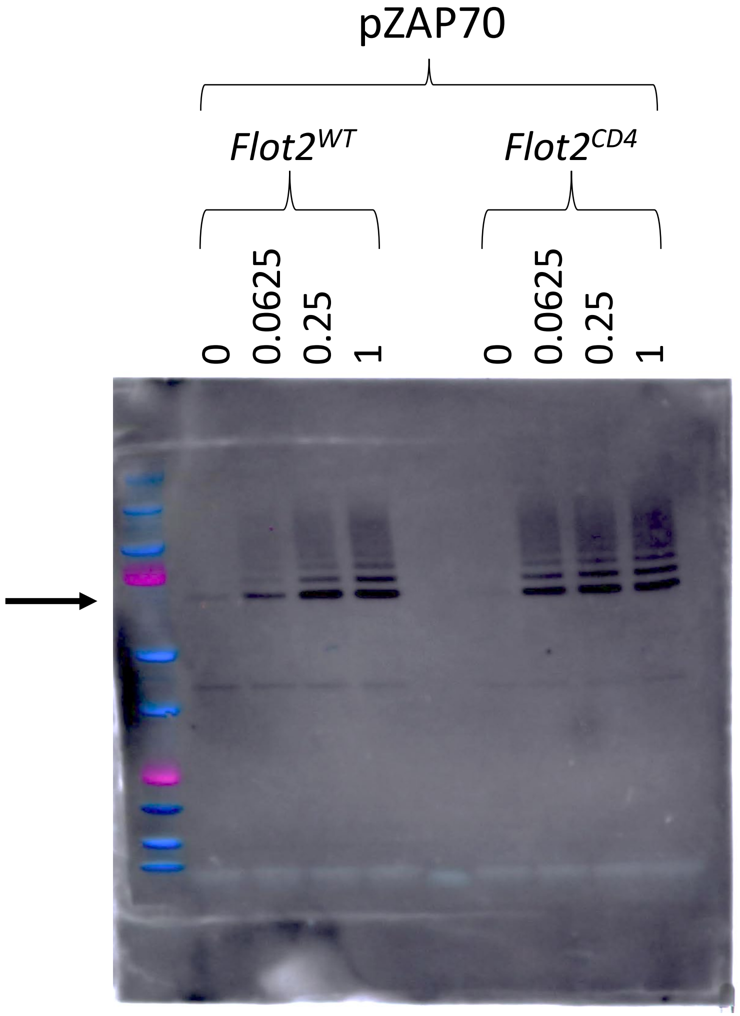

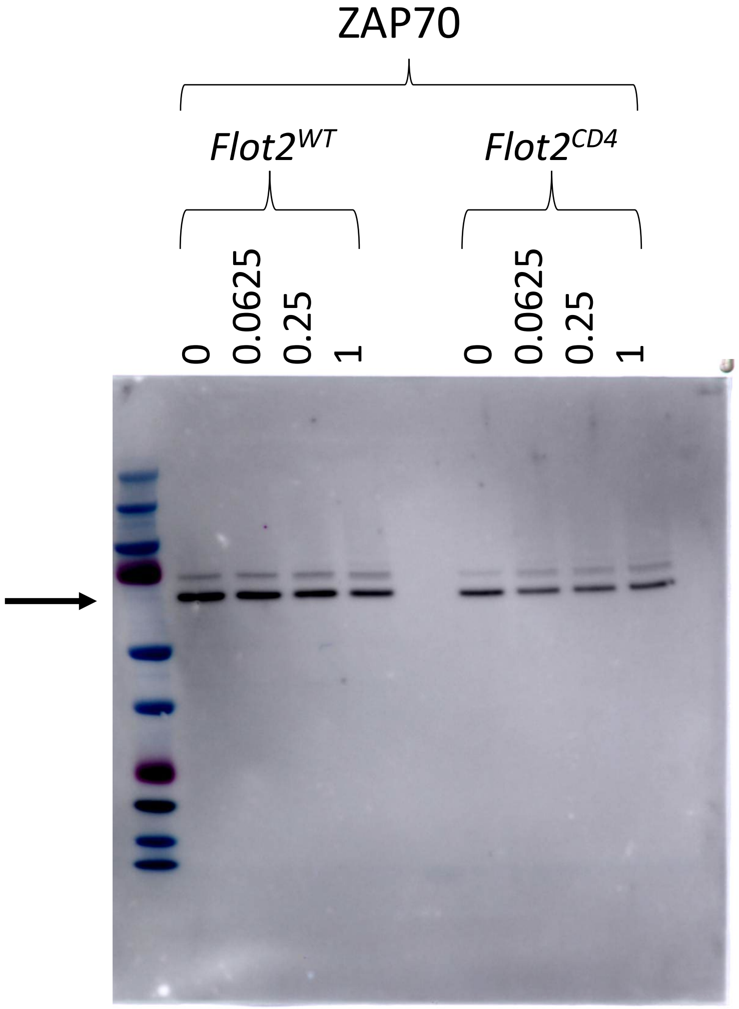

Full unedited gels for Figure 6D

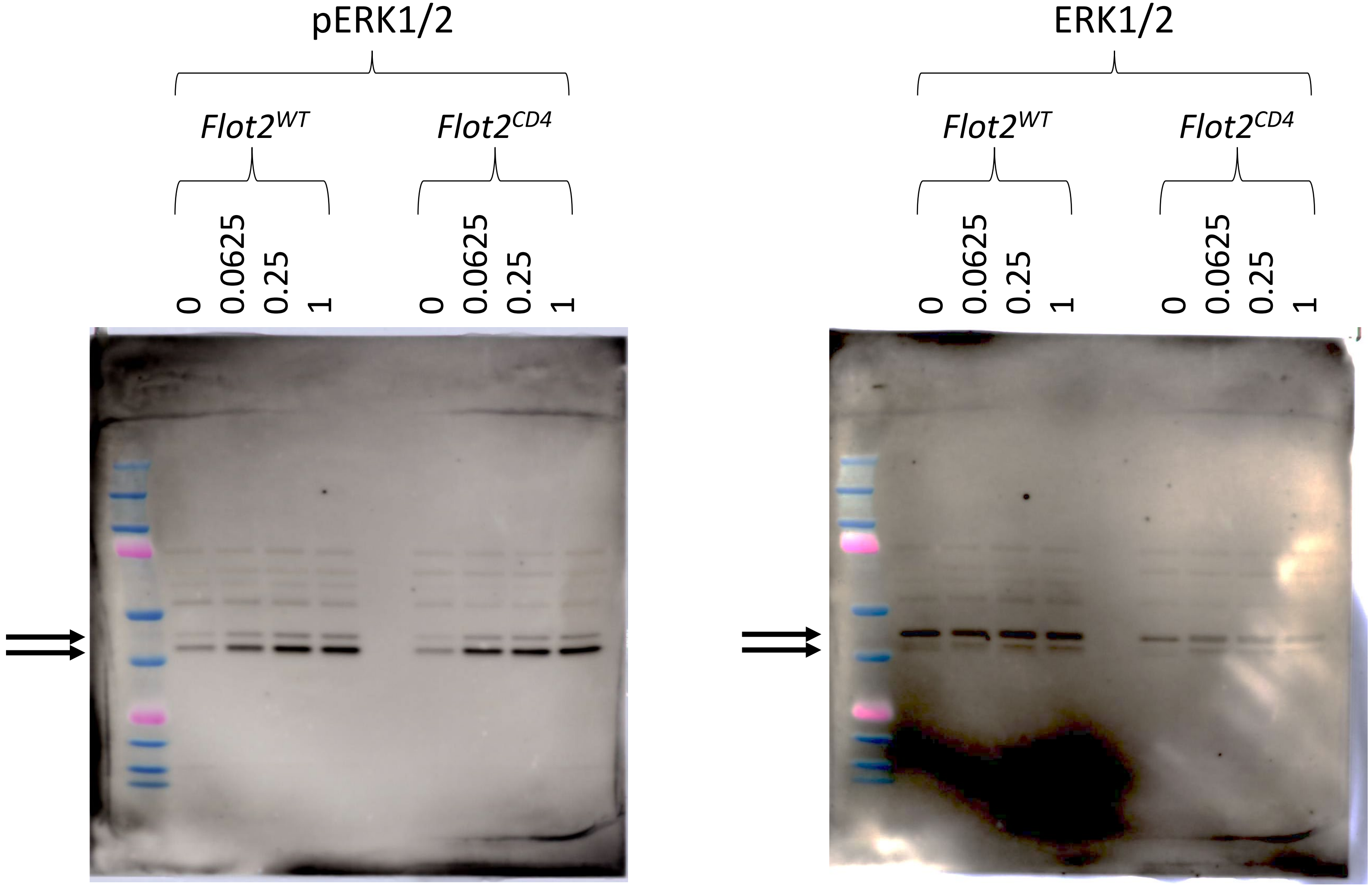

Full unedited gels for Figure 6D

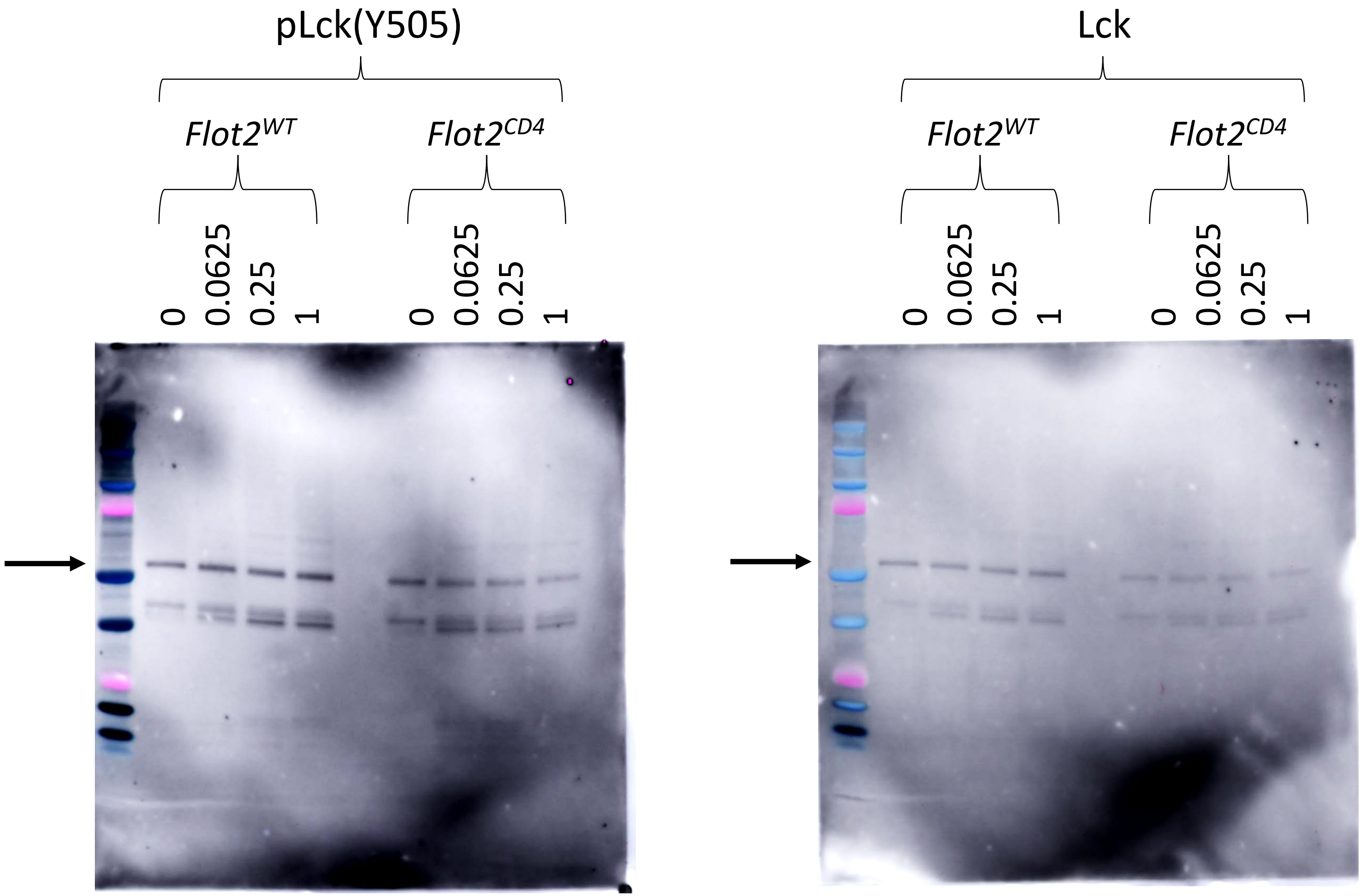

Supplement: Unedited blot and gel images [file jciinsight-9-182328-s009.pdf]
